# Anti-HIV Ermiasolides from *Croton megalocarpus*

**DOI:** 10.3390/molecules27207040

**Published:** 2022-10-19

**Authors:** Ermias Mergia Terefe, Faith Apolot Okalebo, Solomon Derese, Moses K. Langat, Eduard Mas-Claret, Kamal Ahmad Qureshi, Mariusz Jaremko, Joseph Muriuki

**Affiliations:** 1 Department of Pharmacology and Pharmacognosy, School of Pharmacy and Health Sciences, United States International University-Africa, Nairobi P.O. Box 14634-00800, Kenya; 2 Department of Pharmacology and Pharmacognosy, College of Health Sciences, University of Nairobi, Nairobi P.O. Box 30197-00100, Kenya; 3 Department of Chemistry, University of Nairobi, Nairobi P.O. Box 30197-00100, Kenya; 4 Royal Botanic Gardens, Kew, Kew Green, Richmond, Surrey TW9 3AE, UK; 5 Department of Pharmaceutics, Unaizah College of Pharmacy, Qassim University, Unaizah 51911, Saudi Arabia; 6 Smart-Health Initiative (SHI) and Red Sea Research Center (RSRC), Division of Biological and Environmental Sciences and Engineering (BESE), King Abdullah University of Science and Technology (KAUST), Thuwal 23955, Saudi Arabia; 7 Centre for Virus Research, Kenya Medical Research Institute, Nairobi P.O. Box 14634-00800, Kenya

**Keywords:** *Croton megalocarpus*, HIV-1, ermiasolides, anti-HIV activity, cytotoxicity

## Abstract

In recent years, elucidation of novel anti-HIV bioactive compounds from natural products is gaining importance rapidly, not only from the research and publications, but also from controlled clinical studies. Here we report three new anti-HIV eudesmane-type sesquiterpenes, 5β-Hydroxy-8α-methoxy eudesm-7(11)-en-12,8-olide (**1**), 5β,8α-Dihydroxy eudesm-7(11)-en-12,8-olide (**2**) and 5β-Hydroxy-8H-β-eudesm-7(11)-en-12,8-olide (**3**). These are trivially named ermiasolide A-C and were isolated from the bark of *Croton megalocarpus*. 5β-Hydroxy-8α-methoxy eudesm-7(11)-en-12,8-olide (**1**), showed the highest anti-HIV activity by inhibiting 93% of the viral replication with an IC_50_ = 0.002 µg/mL. On the other hand, 5β-Hydroxy-8H-β-eudesm-7(11)-en-12,8-olide (**3**) and 5β,8α-dihydroxy eudesm-7(11)-en-12,8-olide (**2**), inhibited viral replication by 77.5% at IC_50_ = 0.04 µg/mL and 69.5% at IC_50_ = 0.002 µg/mL, respectively. Molecular docking studies showed that the proposed mechanism of action leading to these results is through the inhibition of HIV-protease.

## 1. Introduction

In our continued study of anti-HIV potential of compounds isolated from *Croton* plants, we report three eudesmane sesquiterpenoids from *C. megalocarpus* with modest anti-HIV activities [1,2]. *C. megalocarpus* is an important medicinal plant, with several traditional medicinal uses. Traditionally, *C. megalocarpus* is mainly used to treat respiratory problems, fever [3,4,5] and wounds [5,6]. *C. megalocarpus* is also used in the treatment of constipation [7] and as an herbal medicine for backache, chest problems [8,9], malaria [10,11,12] and stomach ache [8,9,12]. It has also been reported for its medicinal potential, with reported anti-inflammatory, antifungal, antibacterial, antinociceptive, wound healing and molluscidal properties [13]. Thus far, *C. megalocarpus* has only yielded cembrane, labdane, kaurene, clerodane and abietane diterpenoids [14,15,16,17]. In our recent study we reported two previously-undescribed crotofolane diterpenoids: 1β-acetoxy-3β-chloro-5α,6α-dihydroxycrotocascarin L, trivially named ermiasoid, [18], 11β-acetoxycrotocascarin L and the known compound crotocascarin K [1]. In the same study, evidence for anti-HIV activity was found through decrease of HIV replication by inhibition of HIV-1 protease.

In this study we report for the first time sesquiterpene lactones from *C. megalocarpus.* Sesquiterpene lactone is a large subclass of terpenoids often found in plants. Sesquiterpene lactones play an important role in plant defence in nature, acting as antibacterials, antivirals, antifungals, and insecticides [19] and have piqued the interest of researchers in recent years, primarily due to their cytotoxic and anticancer properties [20]. Several eudesmane-type sesquiterpene lactones have displayed cytotoxic and potential antiviral activity. Zhu et al. [21] have demonstrated cytotoxic activity of three eudesmane lactones isolated from *Ajania przewalskii* Poljakov (Asteraceae).

A number of sesquiterpenes have previously been isolated from *Croton* species. β-caryophyllene from *C. aubrevillei, C. geayi* [22]; caryophyllene oxide, γ-cadinene and α-cadinene from *C. geayi* [22]; blumenol A from *C. pedicellatus* [23]; crocrassins A [24], crocrassins B [24], cracroson H [25], 6S-hydroxy-cyperenoic acid [26], crassifterpenoid A [26] from *C. crassifolius,* and patchoulenone [27] from *C. oblongifolius.*

**Figure 1:**
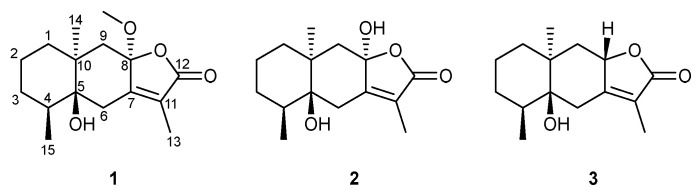
Structures of compounds isolated from *Croton megalocarpus.* Ermiasolide A (**1**); Ermiasolide B (**2**), Ermiasolide C (**3**).

Eudesmane-type sesquiterpenoids were previously reported from Croton species, including C. pseudopulchellus and C. gratissimus [28,29]. However, they were not reported previously from C. megalocarpus, which makes this work interesting. Herein, we report the isolation and anti-HIV activity of three new eudesmane-type sesquiterpenes (Figure 1): 5β-Hydroxy-8α-methoxy eudesm-7(11)-en-12,8-olide (**1**), 5β,8α-Dihydroxy eudesm-7(11)-en-12,8-olide (**2**), and 5β-Hydroxy-8H-β-eudesm-7(11)-en-12,8-olide (**3**) from the stem bark of *C. megalocarpus,* trivially named as ermiasolide A, B and C respectively.

## 2. Results and Discussion

### 2.1. Characterization and Structural Elucidation

**Figure 2:**
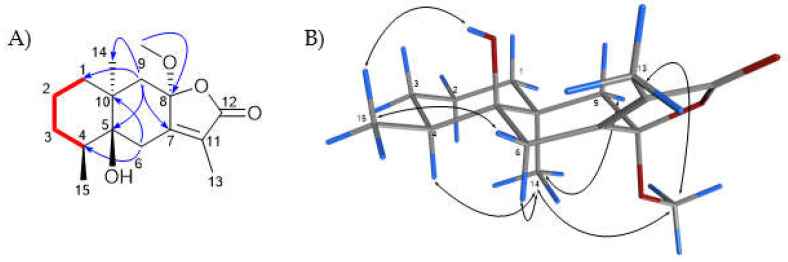
(**A**) Key COSY (red bold bonds) and HMBC (blue arrows) correlations (**B**) NOESY (double headed arrows) correlations observed for compound **1**.

**5β-Hydroxy-8α-methoxy eudesm-7(11)-en-12,8-olide (1):** Compound **1** was isolated as a brown oil. The HRMS showed a molecular ion peak [M + H]^+^ at *m/z* 281.1744 for C_16_H_25_O_4_ (calc. C_16_H_24_O_4_ + H, *m/z* 281.1753). The ^1^H NMR spectrum of **1** (Table 1) showed four methyl proton resonances at δ_H_ 3.13 (s), suggesting methoxy groups, 1.85 (d, 1.3 Hz), 1.24 (s) and 0.90 (d, 6.7 Hz). The ^13^C NMR, DEPT and HSQC-DEPT spectra supported 16 carbon resonances, including a carbonyl resonance at δ_C_ 172.0, two alkene resonances at δ_C_ 156.8 and 127.7, a hemiketal carbon at δ_C_ 106.5, an oxygenated carbon resonance at δ_C_ 77.8, a methoxylated carbon at δ_C_ 50.4 and carbon resonances for three methyls, five methylenes and one methine. The above information suggested that compound **1** was a methoxylated sesquiterpenoid. The use of 2D NMR spectra, HMBC and COSY suggested that an allylic proton resonance at δ_H_ 1.85 (d, 1.3 Hz) was part of an α,β-unsaturated carbonyl system with carbonyl carbon resonance at δ_C_ 172 (C-12) and alkene carbon resonances at δ_C_ 156.8 (C-7) and 127.7 (C-11). A carbon resonance at δ_C_ 106.5 was assigned to the ketal group at C-8 and was observed in the HMBC spectrum to correlate with the methoxy group proton resonance (δ_H_ 3.13, s) and methylene proton resonances assignable to H_2_-6 and H_2_-9. In addition, H_2_-6 proton resonances showed correlations in the HMBC spectrum with C-4, C-5, C-7, C-10 and C-11, whereas H_2_-9 showed correlations in the HMBC spectrum with C-1, C-5, C-7, C-8 and C-14. C-4 was observed to be a methine carbon resonance, which corresponded with a proton resonance at δ_H_ 1.96 (m) for H-4. The H-4 proton resonance was coupled with a methyl doublet proton at δ_H_ 0.90 (d, 6.7 Hz), attributable to H-15. In addition, H-4 was coupled to proton resonances for H_2_-3, which were coupled to H_2_-2 in the COSY spectrum. H_2_-2 was also coupled to H_2_-1 in the COSY spectrum. The above data suggested that compound **1** was a methoxylated eudesmane sesquiterpenoid that was determined to be a eudesmane class. The NOESY spectrum of compound **1**, acquired using DMSO, showed that the methoxy group at C-8, H-4 and 3H-14 were on the same face of the molecule, whereas 5-OH and 3H-15 were on the opposite face of the molecule (Figure 2). The HRMS data [M + H]^+^ _=_ 280.1744 supported the proposed structure; hence, **1** was determined to be undescribed 5β-hydroxy-8α-methoxy eudesm-7(11)-en-12,8-olide, trivially named ermiasolide-A.

**5β,8α-Dihydroxy eudesm-7(11)-en-12,8-olide (2):** Compound **2** was isolated as a yellowish oil. The HRMS spectrum of compound **2** showed a molecular ion peak [M + H]^+^ at *m/z* 267.1589 for C_16_H_22_O_4_ (calc. C_15_H_22_O_4_ + H, *m/z* 267.1591). The ^1^H NMR spectrum (Table 1) of this compound showed three methyl proton resonances at δ_H_ 1.78 (s), 1.29 (s) and 0.89 (d, 6.7 Hz) and was missing the resonance for a methoxy group that was observed in compound **1.** The ^13^C, DEPT and HSQC-DEPT NMR spectra supported 15 carbon resonances, including a carbonyl resonance at δ_C_ 173.0, two alkene resonances at 158.9 and 125.3, a hemiketal carbon at δ_C_ 104.1, an oxygenated carbon resonance at δ_C_ 78.0 and carbon resonances for three methyls, five methylenes and one methine. The ^13^C and DEPT NMR spectra were missing the methoxylated carbon resonance as in compound **1**. The 2D spectra for compound **2** were similar to those of compound **1**. Compound **2** was determined to be an undescribed derivative of compound **1**, 5β,8α-dihydroxy eudesm-7(11)-en-12,8-olide, trivially named ermiasolide B.

**Figure 3:**
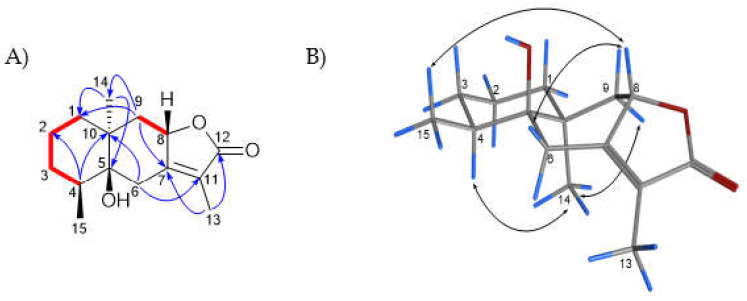
(**A**): Key COSY (red bold bonds) and HMBC (blue arrows) correlations (**B**): NOESY (double headed arrows) correlations observed for compound **3**.

**5β-Hydroxy-8H-β-eudesm-7(11)-en-12,8-olide (3):** Compound **3** was isolated as a yellowish oil. Compound **3** was determined to be an undescribed 5β-hydroxy-8H-β-eudesm-7(11)-en-12,8-olide, an 8-H derivative of compounds **2** and **1**. HRMS showed a molecular ion peak at *m/z* [M + H]^+^ 251.1641 for C_15_H_22_O_3_ (calc. C_15_H_22_O_3_ + H, *m/z* 251.1642), consistent with **3**. The ^1^H NMR spectrum (Table 1) of this compound showed three methyl proton resonances at δ_H_ 1.80 (s), 0.90 (d) and 0.82 (s) and was missing the resonance for a methoxy group that was observed in compound **1**. The ^13^C, DEPT and HSQC-DEPT NMR spectra supported 15 carbon resonances, including a carbonyl resonance at δ_C_ 175.4, two alkene resonances at δ_C_ 161.4 and 121.5, an oxygenated carbon resonance at δ_C_ 76.5, and carbon resonances for three methyls, five methylenes and two methines. The ^13^C and DEPT NMR spectra were missing the methoxylated carbon resonance present in compound **1**, and only had one oxygenated carbon when compared to **2**. The 2D spectra for compound **3** were similar to those of compounds **1** and **2**. The NOESY spectrum of compound **3** showed that there were NOESY correlations between H-8 and 3H-15, and between H-8 and H-6β, confirming that H-8 was β. There were also NOESY correlations between H-4 and 3H-14, and between 3H-14 and H-9α, similar to what was seen for Compound **1**, confirming they were at the same side of the molecule (Figure 3). Compound **3** was determined to be an undescribed derivative of compounds **1** and **2**, 5β-hydroxy-8H-β-eudesm-7(11)-en-12,8-olide, trivially named ermiasolide C.

### 2.2. In Vitro Cytotoxicity and Anti-HIV Assay

**Figure 4:**
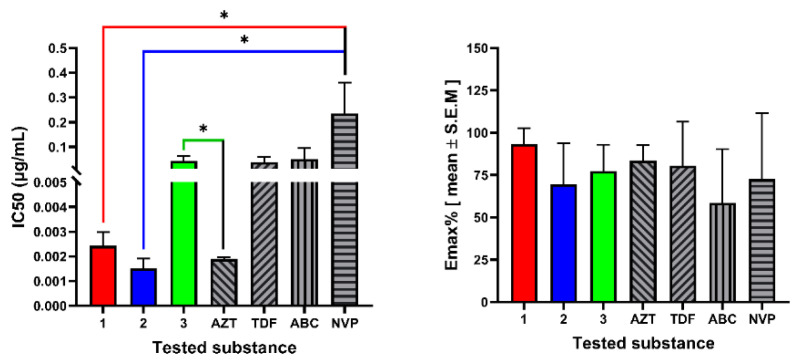
Anti-HIV activity of ermiasolides from *C. megalocarpus.* The results are expressed as the mean of three independent experiments ± S.E.M. AZT, zidovudine; TDF, tenofovir; ABC, abacavir; NVP, nevirapine; 1, ermiasolide A; 2, ermiasolide B; 3, ermiasolide C; MNTC, maximum nontoxic concentration; CC_50_, 50% cytotoxic concentration; EmaxC, maximum cytotoxic effect %; IC_50_, 50% antiviral effect concentrations; EmaxAV, maximum antiviral effect %; SI, selective index. C; control, ns, not significant, * Denotes *p* value < 0.05.

**Figure 5:**
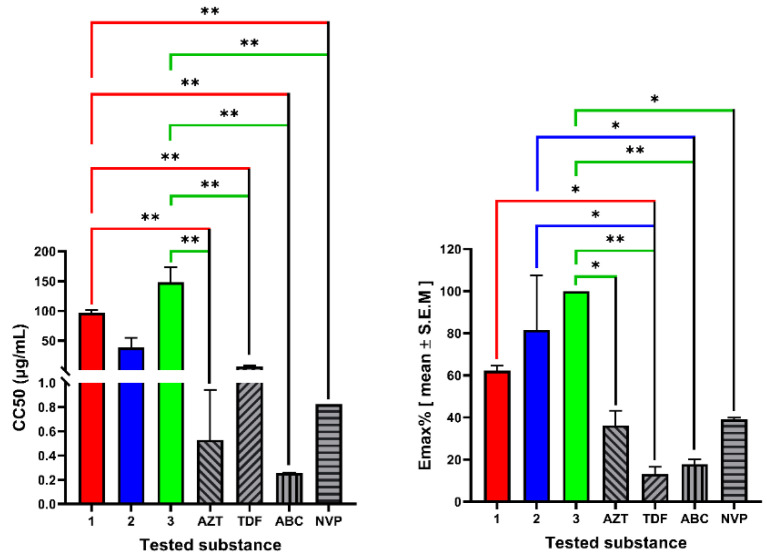
Cytotoxicity of ermiasolides from *C. megalocarpus.* The results are expressed as the mean of three independent experiments ± S.E.M. 1, ermiasolide A; 2, ermiasolide B; 3, ermiasolide C; AZT, zidovudine, ABC, abacavir, NVP, nevirapine; TDF, tenofovir; C, control; ns; not significant, * Denotes *p* value < 0.05, ** Denotes *p* value <0.01.

The cytotoxic and antiviral activity findings for the compound **1–3** are summarized in Table 2. Among the tested compounds, compound **1**, ermiasolide A, displayed the highest anti-HIV activity by inhibiting HIV-induced CPE by 93% at an IC_50_ value of 0.002 µg/mL. Similarly, compound **3**, inhibited viral replication by 77.5% at an IC_50_ value of 0.04 µg/mL (Figure 4). In the MTT cytotoxic assay using human T-lymphocytic MT-4 cells, the compounds showed significantly (*p* < 0.001) higher CC_50_ values than the control drugs, except for compound **2**, which showed CC_50_ values not significantly different from those of the control drugs (Figure 5). The compounds (**1–3**) inhibited viral replication at much lower concentrations (IC_50_) than the maximum non-cytotoxic concentration (MNTC), indicating the safety of the compounds (Table 2).

### 2.3. Assay of Anti-HIV Activity In Silico

A molecular docking study was conducted to predict the binding geometries of the compounds with HIV-1 Reverse Transcriptase and HIV-1 Protease. Table 3 and Table 4 summarize the docking results for the pure compounds and FDA-approved antiretroviral drugs. The predicted free energy of binding obtained for the pure compounds against HIV-1 RT (PDB ID: 1JLB) was higher than the known inhibitor nevirapine (ΔG −7.679 kcal/mol). Similarly, the compounds analyzed also gave higher predicted binding energies than the known inhibitor Atazanavir when the docking was performed on HIV-1 PR (PDB ID: 3EL9 (Figure S1). However, the docked compounds displayed strong binding affinity with HIV-1 protease enzyme compared with HIV-1 reverse transcriptase. The results obtained could indicate that the mechanism of action leading to the observed anti-HIV activity is through HIV-1 protease inhibition, which agrees with the results obtained in vitro.

To better understand the mode of action of the compounds, ligand-protein interactions were visualized. Compound **1** binds to the protease active site with an estimated free energy of binding of −6.067 kcal/mol. The main interactions contributing to this binding energy were the hydrogen bond between Asp-*B*29 and Arg-*A*8 with the lactone carbonyl at C-12 (Figure S2). The methyl group at C-4 and the two cyclohexane rings were involved in hydrophobic interactions with Ala-*B*28, Ile-*B*50 and Val-*B*84, and the methoxy group at C-8 showed hydrogen bonding with Asp-*B*25.

This led us to the hypothesis that the high anti-HIV activity of Ermiasolide A (**1**) and Ermiasolide B (**2**) in vitro could be due to the presence of hydrogen bonding between the methoxy or hydroxy group at C8 and Asp-*B*25. These functional groups were missing in Ermiasolide C (**3**) which could explain its lower antiviral efficacy (Emax_AV_= 77.46 %). Furthermore, the presence of a methoxy group in **1** at C-8 could contribute to its higher anti-HIV activity due to additional hydrophobic interactions with the enzyme active site, as depicted in the in silico study (Figure S2). Previous studies have confirmed the role of hydrophobic interactions as driving forces of ligand–protein interaction [30]. The results obtained could indicate that the mechanism of action leading to the observed anti-HIV activities is through HIV-1 protease inhibition, which agrees with the data obtained in the in vitro assay.

## 3. Experimental Section

### 3.1. Plant Material

The stem bark of *C. megalocarpus* was collected from the United States International University-Africa (USIU) Botanical Garden in June 2020. Ethical approval was obtained from the Kenyatta National Hospital-University of Nairobi Ethics and Research Committee (KNH-UON ERC), approval number KNH/ERC/A/154. Taxonomic identification was done by expert botanist Ms. Lucy M. Wambui and taxonomist Mr. Patrick B. Mutiso of the University of Nairobi, and a voucher specimen (TEREFE E. /044) was deposited at the USIU herbarium for future reference. The extraction and isolation of the plant was carried out as previously described [2,18,19].

### 3.2. Isolation of Compounds ***1***–***3***

Compounds were isolated as previously described [2,18,19]. Briefly, 5 kg of air-dried stem bark of *C. megalocarpus* was extracted using 1:1 CH_2_Cl_2_:CH_3_OH by standing at room temperature, to give 0.33 kg of a solid. This extract was resuspended in water and extracted using hexane, CH_2_Cl_2_, EtOAc and then MeOH (masses 11 g, 8 g, 40 g and 11 g, respectively). The EtOAc extract was chromatographed on silica gel by a step gradient elution initially with 100% CH_2_Cl_2_, followed by 5% EtOAc addition to yield several fractions of 75 mL each. Fractions 12, 17 and 22 gave compounds **1**, **3** and **2,** respectively, on further purification using an isocratic system of 9:1 CH_2_Cl_2_:EtOAc.

### 3.3. Structural Elucidation of Isolated Compounds

The isolated purified compounds were characterized by nuclear magnetic resonance (NMR) and high-resolution mass spectrometry (HRMS) at the Jodrell Laboratory, Royal Botanic Gardens Kew (UK). A Perkin-Elmer Frontier/Spotlight 200 spectrometer was used to collect FTIR spectra. In addition, 1D and 2D NMR spectra in CDCl_3_ were collected at room temperature using a 400 MHz Bruker AVANCE NMR spectrometer. Chemical shifts (δ) were measured in ppm and referenced to the solvent resonances for ^1^H and ^13^C NMR for CDCl_3_ at δ_H_ 7.26 and C 77.23 ppm.

LCMS data were collected using a Vanquish UHPLC system (Thermo Scientific, Waltham, MA, USA) linked to a 100 Hz photodiode array detector (PDA) and a Thermo Scientific Orbitrap Fusion Tribrid (Thermo Scientific) high-resolution tandem mass spectrometer. Chromatographic separation (5 µL) was performed on a Luna C18 column (150 mm × 3 mm i.d., 3 μm, Phenomenex, Torrance, CA, USA) using a mobile phase gradient of 0:90:10 to 90:0:10 (MeOH (A) water, (C) acetonitrile + 1% formic acid (D)) over 60 min. Then, 90% A was held for 10 min and returned to initial conditions over 5 min at 30 °C (flow rate: 400 μL/min). UV detection was done between 210 and 550 nm.

Mass spectrometry detection was performed in positive and negative ionization modes using the full scan and data-dependent MS^2^ and MS^3^ acquisition modes. Total ion current (TIC) chromatograms were obtained over the range of 125–1800 *m/z* using spray voltages of +3.5 kV and −2.5 kV for the positive and negative ionization modes, respectively. Four different scan events were recorded for each ionization mode as follows: (1) full scan; (2) MS^2^ of the most intense ion in scan event 1; (3) MS^3^ of the most intense ion in scan event 2; and (4) MS^3^ of the second most intense ion in scan event 2. Additional parameters for the mass spectrometer included: full scan resolution, 60,000 FWHM; capillary temperature, 350 °C; ion transfer tube temperature, 325 °C; RF lens (%), 50; automatic gain control (AGC) target, 4.0e5 (Full scan) and 1.0e4 (MS^n^); intensity threshold, 1.0e4; CID collision energy, 35; activation Q, 0.25; and isolation window (*m/z*), 4. Nitrogen was used as the drying, nebulizer and fragmentation gas.

### 3.4. Cell Culture, Maintenance and Viability Test

In this study, human T-lymphocyte MT-4 cells (ARP-120) were obtained from the National Institute of Health (NIH) HIV Reagent Program, Division of AIDS, National Institute of Allergy and Infectious Diseases (NIAID), NIH; donated by Dr. Douglas Richman. Human T-lymphocytic MT-4 cells express CD4, CXCR4 and CCR5 and are used for cytotoxicity inhibition assays for antiviral drugs [31,32,33,34]. The vials containing frozen MT-4 cells were retrieved from the liquid nitrogen tank and rapidly thawed [33]. In a tissue culture laminar flow hood, the exterior of the vials was decontaminated by spraying 70% ethanol. Then, the vial was opened and resuspended in 10 mL complete culture medium (CCM) containing RPMI-1640 with 2 mM L-glutamine supplemented with 10% fetal bovine serum (FBS), 10 mM HEPES buffer, 100 IU of penicillin/mL, 100 μg of streptomycin/mL, 1 ng of recombinant interleukin-2 (Invitrogen)/mL and 2 μg of phytohemagglutinin/mL as described previously by Gao et al. [33]. The CCM was sterilized with a 0.22 µm membrane filter before use. The cultures were incubated at 37 °C in a humidified incubator with atmospheric conditions of 5% CO_2_. Every 3–5 days, the cultures were centrifuged, pelleted and resuspended in 5–10 mL RPMI 1640 supplemented with 10% FBS. The cell count and viability were measured by the trypan blue dye exclusion technique [34].

### 3.5. Cytotoxicity Testing

The cytotoxicity test was conducted to evaluate the cytotoxicity of the plant extracts by measuring cell death caused by the plant extracts. The assay was conducted using an MTT colorimetric assay as described by [2,19,31,35,36]. The MTT assay is based on the reduction of the yellow-colored tetrazolium salt MTT 3-(4,5-dimethylthiazol-2-yl)-2,5-diphenyltetrazolium bromide) by NAD(P)H-dependent cellular oxidoreductase enzymes [37] to an insoluble dark-blue colored formazan that can be measured spectrophotometrically [38]. Formazan production indicates the number of viable cells; therefore, an increase or decrease in cellular viability results in a change in the amount of formazan formed, which indicates the degree of cellular cytotoxicity (CC_50_) caused by the plant extract.

MTT (3-(4,5-dimethylthiazol-2-yl)-2,5-diphenyl tetrazolium bromide) was dissolved in PBS to obtain a final concentration of 5 mg/mL and filtered to sterilize and remove insoluble residue [35,36]. The assay was carried out in 96-well, flat-bottomed microtiter plates. To each well, 200 µL of MT-4 cells (1 × 10^5^ cells) in growth media was added. The plates were preincubated for 24 h at 37 °C to allow stabilization. Then, 50 μL of the test compounds (at a concentration of 4 mg/mL) were added to the first column of the well. With a multichannel pipette, 50 μL was transferred (in triplicate) from the wells labeled 1 to wells labeled 2, and such transfers were continued (serial dilution), moving from left to right, changing tips prior to mixing contents of the next column of wells. Finally, 50 μL was discarded from the wells in column 12. Different concentrations (800–8.192 × 10^5^ μg/mL) of test compounds were prepared through serial dilution. Each dilution was tested in triplicate. The negative control (NC) wells contained 50 µL of MT-4 cells in 0.5% DMSO [39]. Positive control (zidovudine, tenofovir, abacavir and nevirapine) drugs were also added in triplicate.

A 96-well microtiter plate containing the test compounds, positive and negative controls was incubated at 37 °C in a humidified atmosphere of 5% CO_2_ for 5 days. After incubation, 20 μL of MTT reagent (5 mg/mL MTT in phosphate-buffered saline) was added to each test well and control well. The plate was further incubated at 37 °C in a CO_2_ incubator for 4 h. After 4 h of incubation, 100 μL of DMSO was added to dissolve the dark-blue formazan crystals from surviving cells [40]. After the formazan crystals were dissolved completely, the resulting optical density (OD) readings were measured relative to the controls on an ELISA plate reader at 570 nm at a reference wavelength of 620 nm [35]. The 50% cellular cytotoxicity concentration (CC_50_) was defined as the concentration of the test compound that reduced the absorbance of the negative control by 50%. A dose-response curve was plotted to enable the calculation of the concentrations that reduced the number of viable cells by 50% (CC_50_). The concentration that determined cell viability above 80% (CC_20_) was chosen as the maximum non-toxic concentration (MNTC).

### 3.6. Viral Culture

Human immunodeficiency virus type 1 (HIV-1_IIIB_) strain was obtained from the NIH HIV Reagent Program, Division of AIDS, NIAID, NIH. Dr. Robert Gallo donated human immunodeficiency virus-1 IIIB (ARP-398). HIV-1_IIIB_ is highly capable of replicating in human T cell lines and appears to be well adapted for in vitro culture in T cells [41]. The frozen HIV-1_IIIB_ vials were thawed by immersion in room temperature water, and the vial was swirled until viruses were thawed. Then, 400 µL of the virus was transferred to 75 cm^2^ tissue culture flasks (T75) containing 10 mL of 1 × 10^5^ pelleted MT4 cells/mL, vortexed and incubated at 37 °C for 1 hr for virus adsorption. After 1 hr, 20 mL of RPMI-1640 was added gently and mixed with vortexing. The cells were then pelleted by centrifuging at 1500 rpm for 5 min. The pelleted cells were then washed and resuspended in 30 mL RPMI 1640 with L-glutamine and 20% fetal bovine serum in a T75 flask, incubated at 37 °C and monitored for the development of cytopathic effects (CPEs). The MT-4 cells inoculated with HIV showed markedly different cell growth and viability patterns than mock-infected cells. The number of viable cells rapidly decreased at 24 h post-infection, and by day 4, only 2–4% of the infected MT-4 cells were viable. In contrast, the mock-infected cells grew well, reaching a plateau between days 2 and 4. After 4 days, the viability of these cells began to decline appreciably. The infected cells displayed cytopathic effects (CPE), including becoming round, losing their surface characteristics, becoming refractile and diminishing in size. By day 3, many infected cells developed balloon-like, cytoplasmic swelling. These observations agreed with previous reports of many scholars who used the MT-4 cell line to evaluate the anti-HIV activity of various compounds [2,36,42,43,44].

The virus infectivity test was performed to determine the infectious titer of the virus, which can cause cytopathic effects (CPE) in tissue culture over a reasonable period of 3 to 10 days while cells in culture remain viable [31,37]. In addition, this procedure was performed to quantify the amount of virus required to produce a cytopathic effect in 50% of cell culture replicates (TCID_50_) [35]. CPE induced by the virus was observed under a microscope after 3–4 days. The TCID_50_ was then calculated as described by [2,19]. For the anti-HIV activity testing, 640 µL of HIV-1_IIIB_ virus at 1.26 × 10^8^ TCID_50_/mL was used to infect 1 × 10^8^ cell/mL. All procedures were carried out following biosafety guidelines defined in the BMBL, NIH-CDC HHS Publication No. (CDC) 21-1112 [44].

### 3.7. Anti HIV Activity Testing

The effects of the test compounds in preventing cytopathic effects that occur as a result of HIV-1 replication were evaluated by MTT colorimetric assay. MT-4 cells suspended at 1 × 10^8^ cells/mL were infected with 640 µL of HIV-1_IIIB_ virus at 1.26 × 10^8^ TCID_50_/mL as described in above. After infection, 200 µL HIV-infected MT-4 cells (1 × 10^5^ cells/well) in growth media were added to each well. The plates were preincubated for 24 h at 37 °C to allow stabilization. Then, 50 μL of the test compounds (at a concentration of 4 mg/mL) were added to the first column of the well. With a multichannel pipette, 50 μL was transferred (in triplicate) from the wells labeled 1 to wells labeled 2. Such transfers were continued (serial dilution), moving from left to right, changing tips prior to mixing contents of the next column of wells. Finally, 50 μL was discarded from the wells in column 12. Different concentrations (800 to 8.192 × 10^5^ μg/mL) of test compounds were prepared through serial dilution. Each dilution was tested in triplicate. The microtiter plates were incubated at 37 °C in a 5% CO_2_ incubator for 5 days. Two negative controls, infected untreated cells, and uninfected untreated (mock) cells and four positive controls (zidovudine, tenofovir, abacavir and nevirapine) were also included. After 5 days of incubation, cell viability was determined by the MTT assay as described by [2,19,32,38,39,45,46].

A dose-response curve was plotted to calculate the concentrations that reduced viral replication by 50% (IC_50_) [45,46]. The effective inhibitory concentration at 50% (IC_50_) is defined as the concentration of the test compound that achieves 50% protection in infected cultures. The efficacy of the test compounds, was determined by calculating the viral inhibition rate (Emax_AV_) using Equation (1), as previously described by [43,47].

(1) $$Viral\;inhibition\;rate\;\left(Emax_{AV}\right)=\frac{{\left(OD_{T}\right)}_{HIV}-{\left(OD_{C}\right)}_{HIV}}{{\left(OD_{C}\right)}_{mock}-{\left(OD_{C}\right)}_{HIV}}\;\times \;100\;$$

where, ${\left(OD_{T}\right)}_{HIV}$ is the optical density measured at a given concentration of the test compound in HIV-infected cells; ${\left(OD_{C}\right)}_{HIV}$ is the optical density measured for the negative control infected untreated cells; ${\left(OD_{C}\right)}_{mock}$ is the optical density measured for the negative control uninfected untreated cells. The selectivity index (SI) of the test compounds was calculated using Equation (2), as the ratio of 50% cytotoxic concentration (CC_50_) to 50% effective concentration (IC_50_). Thus, SI reflects both the antiviral activity and eventual toxicity of the test compounds. Thus, a high SI value indicates low toxicity of the test compound and high activity against the virus.

(2) $$\mathrm{Selectivity}\;\mathrm{index}\;\left(\mathrm{SI}\right)=\frac{50\%\;\mathrm{cytotoxic}\;\mathrm{concentration}\;\left(\mathrm{CC}50\right)}{50\%\;\mathrm{Inhibitory}\;\mathrm{concentration}\;\left(\mathrm{IC}50\right)}$$

### 3.8. Docking Experiments

The docking studies were performed on MOE2015 software package using HIV-1 RT in complex with known inhibitor Nevirapine (PDB ID: 1JLB) and wild-type HIV-1 protease complexed with known antiviral atazanavir (PDB ID: 3EL9). The proteins were prepared by first removing all water molecules, and in the case of protease also the sulfate and formate ions present in the PDB file. Then, hydrogens were added, and the structures were protonated. For the ligands, a first optimization step was performed on **1** and **3** using molecular mechanics forcefield MMFF94x and optimized using the DFT method at the B3LYP/6–31G* level (gas phase) using Gaussian09. To validate the docking protocol used, known inhibitors Nevirapine and Saquinavir were removed from their corresponding binding pockets and redocked. The Root Mean Square Deviation (RMSD) values from the known co-crystallized conformation were 0.4421 Å and 1.6038 Å, respectively. Compounds **1–3** were docked using MOE2015 with triangle matcher, scoring by London dG, 30 poses as placement method and rigid receptor, GBVI/WSA dG 5 poses as refinement method in both targets. The docking procedure was repeated in three independent runs. The lowest scoring affinity pose in each ligand was used to study the ligand interactions.

### 3.9. Statistical Analysis

The CC_50_ and IC_50_ values were calculated with GraphPad Prism v9 using the equation for sigmoidal dose-response (variable slope). Statistical significance in the comparison between control drugs and extract cytotoxicity and antiviral activity parameters (CC_50_, Emax_C_, IC_50_ and Emax_AV_) were determined by one-way ANOVA followed by Dunnett’s post hoc tests. A difference was considered significant when *p* < 0.05.

## 4. Conclusions

The present study has shown that *C. megalocarpus* has antiviral compounds in addition to other biologically active compounds. Specifically, eudesmane-type sesquiterpenes 5β-Hydroxy-8α-methoxy eudesm-7(11)-en-12,8-olide (**1**), 5β,8α-Dihydroxy eudesm-7(11)-en-12,8-olide (**2**) and 5β-Hydroxy-8H-β-eudesm-7(11)-en-12,8-olide (**3**) from *C. megalocarpus* have been found, characterized and promising anti-HIV activity discovered. These findings hint that members of this plant family may provide compounds for new anti-virals for treating HIV.

## Figures and Tables

**Table 1:** NMR data for the three isolated ermiasolides from *C. megalocarpus* in CDCl_3_ (*J* in Hz).

	1		2		3	
No.	^13^C NMR	^1^H NMR	^13^C NMR	^1^H NMR	^13^C NMR	^1^H NMR
1α	34.3	1.52	34.2	1.55 m	36.9	1.50 m
1β		1.14		1.14 m		1.34 m
2α	20.0	1.67 m	20.0	1.68 m	21.2	1.67 m
2β		1.59 m		1.49 m		1.59 m
3α	30.1	1.48 m	30.0	1.48 m	30.2	1.50 m
3β		1.30 m		1.27 m		1.23 m
4	34.1	1.96 m	34.1	1.94 m	36.2	1.81 m
5	77.8	-	78.0	-	76.5	-
6α	32.0	2.71 d (13.9)	31.8	2.71 d (13.9)	35.4	2.55 m
6β		2.27 dd (1.4, 13.9)		2.43 d (13.9)		2.55 m
7	156.8	-	158.9	-	161.4	-
8	106.5	-	104.1	-	78.0	5.44 m
9α	45.8	1.96 d (13.7)	47.0	1.90 m	43.2	2.24 dd (13.6, 11.2)
9β		1.82 d (13.7)		1.88 m		1.39 dd (5.7, 13.6)
10	38.9	-	38.9	-	38.5	-
11	127.7	-	125.3	-	121.5	-
12	172.0	-	173.0	-	175.4	-
13	8.6	1.85 d (1.3)	8.4	1.78 s	8.6	1.80 s
14	21.0	1.24 s	21.2	1.29 s	23.9	0.82 s
15	15.3	0.90 d (6.7)	15.2	0.89 d (6.7)	14.8	0.93 d (6.7)
OCH_3_	50.4	3.16 s				

**Table 2:** Cytotoxicity and anti-HIV-1 activities of ermiasolides from *C. megalocarpus*.

Materials	Cytotoxicity			Antiviral Activity		SI
	MNTC (µg/mL)	CC_50_ (µg/mL)	Emax_C_ (%)	IC_50_ (µg/mL)	Emax_AV_ (%)	
**FDA approved antiretroviral drugs**						
**AZT**	0.38 ± 0.19	0.53 ± 0.29	36.28 ± 0.83	0.002 ± 0.00	83.5 ± 0.57	279.4
**TDF**	4.92 ± 0.71	6.73 ± 0.24	13.17 ± 0.43	0.04 ± 0.01	80.55 ± 0.46	176.5
**ABC**	0.18 ± 0.03	0.26 ± 0.00	17.83 ± 0.57	0.05 ± 0.031	58.67 ± 0.43	5.0
**NVP**	0.57 ± 0.0	0.82 ± 0.0	39.13 ± 0.65	0.24 ± 0.09	72.53 ± 0.47	3.5
**Ermiasolides isolated from *C. megalocarpus***						
1	41.84 ± 0.11	96.77 ± 0.44	62.21 ± 0.67	0.002 ± 0.00	93.4 ± 0.60	39,872.3
2	4.973 ± 0.25	38.44 ± 0.63	81.59 ± 0.41	0.002 ± 0.00	69.51 ± 0.26	25,339.5
3	64.93 ± 0.26	148.1 ± 0.05	65.00 ± 0.01	0.044 ± 0.01	77.46 ± 0.93	3384.4

Results are shown as means ± S.E. M (*n* = 3). AZT, zidovudine; TDF, tenofovir; ABC, abacavir; NVP, nevirapine; 1, ermiasolide A; 2, ermiasolide B; 3, ermiasolide C; MNTC, maximum nontoxic concentration; CC_50_, 50% cytotoxic concentration; Emax_C_, maximum cytotoxic effect %; IC_50_, 50% antiviral effect concentrations; Emax_AV_, maximum antiviral effect %; SI, selective index.

**Table 3:** Molecular docking analysis of against HIV-1 reverse transcriptase in complex with Nevirapine (PDB ID: 1JLB).

Code	Name of Compounds	Free Energy of Binding (ΔG) kcal/mol
NVP	Nevirapine	−7.679
1	5β-Hydroxy-8α-methoxy eudesm-7(11)-en-12,8-olide	−2.317
2	5β,8α-Dihydroxy eudesm-7(11)-en-12,8-olide	−2.839
3	5β-Hydroxy-8H-β-eudesm-7(11)-en-12,8-olide	−3.555

**Table 4:** Molecular docking analysis of against HIV-1 protease in complex with Atazanavir (3EL9).

Code	Name of Compounds	Free Energy of Binding (ΔG) kcal/mol
ATV	Atazanavir	−10.437
1	5β-Hydroxy-8α-methoxy eudesm-7(11)-en-12,8-olide	−6.067
2	5β,8α-Dihydroxy eudesm-7(11)-en-12,8-olide	−5.994
3	5β-Hydroxy-8H-β-eudesm-7(11)-en-12,8-olide	−5.850

## Supplementary Materials

The following supporting information can be downloaded at: https://www.mdpi.com/article/10.3390/molecules27207040/s1. The HRMS, and NMR spectra of the compounds are available in the Supplementary Information.
