# Supplementary material for: Anti-HIV Ermiasolides from Croton megalocarpus
Source: Molecules. 2022 Oct 19;27(20):7040. doi: 10.3390/molecules27207040 (PMC9610617; doi:10.3390/molecules27207040)

molecules

Supplementary information

Anti-HIV Ermiasolides from *Croton megalocarpus*

Ermias Mergia Terefe [1,2,*], Faith Apolot. Okalebo [2], Solomon Derese [3], Moses K. Langat [4], Eduard Mas-Claret [4] Kamal Ahmad Qureshi [5], Mariusz Jaremko [6], and Joseph Muriuki [7]

[1] Department of Pharmacology and Pharmacognosy, School of Pharmacy and Health Sciences, United States International University-Africa, Nairobi P.O. Box 14634–00800, Kenya; eterefe@usiu.ac.ke (E.M.T)
[2] Department of Pharmacology and Pharmacognosy, College of Health Sciences, University of Nairobi, Nairobi P.O. Box 30197-00100, Kenya; faith.okalebo@uonbi.ac.ke (F.A.O)
[3] Department of Chemistry, University of Nairobi, Nairobi P.O. Box 30197-00100, Kenya; sderese@uonbi.ac.ke (S.D.)
[4] Royal Botanic Gardens, Kew, Kew Green, Richmond, Surrey TW9 3AE, UK e.mas-claret@kew.org (E.M.-C.); m.langat@kew.org (M.K.L.)
[5] Department of Pharmaceutics, Unaizah College of Pharmacy, Qassim University, Unaizah 51911, Saudi Arabia; ka.qurishe@qu.edu.sa (K.A.Q)
[6] Smart-Health Initiative (SHI) and Red Sea Research Center (RSRC), Division of Biological and Environmental Sciences and Engineering (BESE), King Abdullah University of Science and Technology (KAUST), Thuwal 23955, Saudi Arabia; mariusz.jaremko@kaust.edu.sa (M.J.)
[7] Centre for Virus Research, Kenya Medical Research Institute, Nairobi, Kenya; jmuriuki@kemri.org (J.M)
* Correspondence: eterefe@usiu.ac.ke or mergiae@gmail.com; Tel.: +254746272742

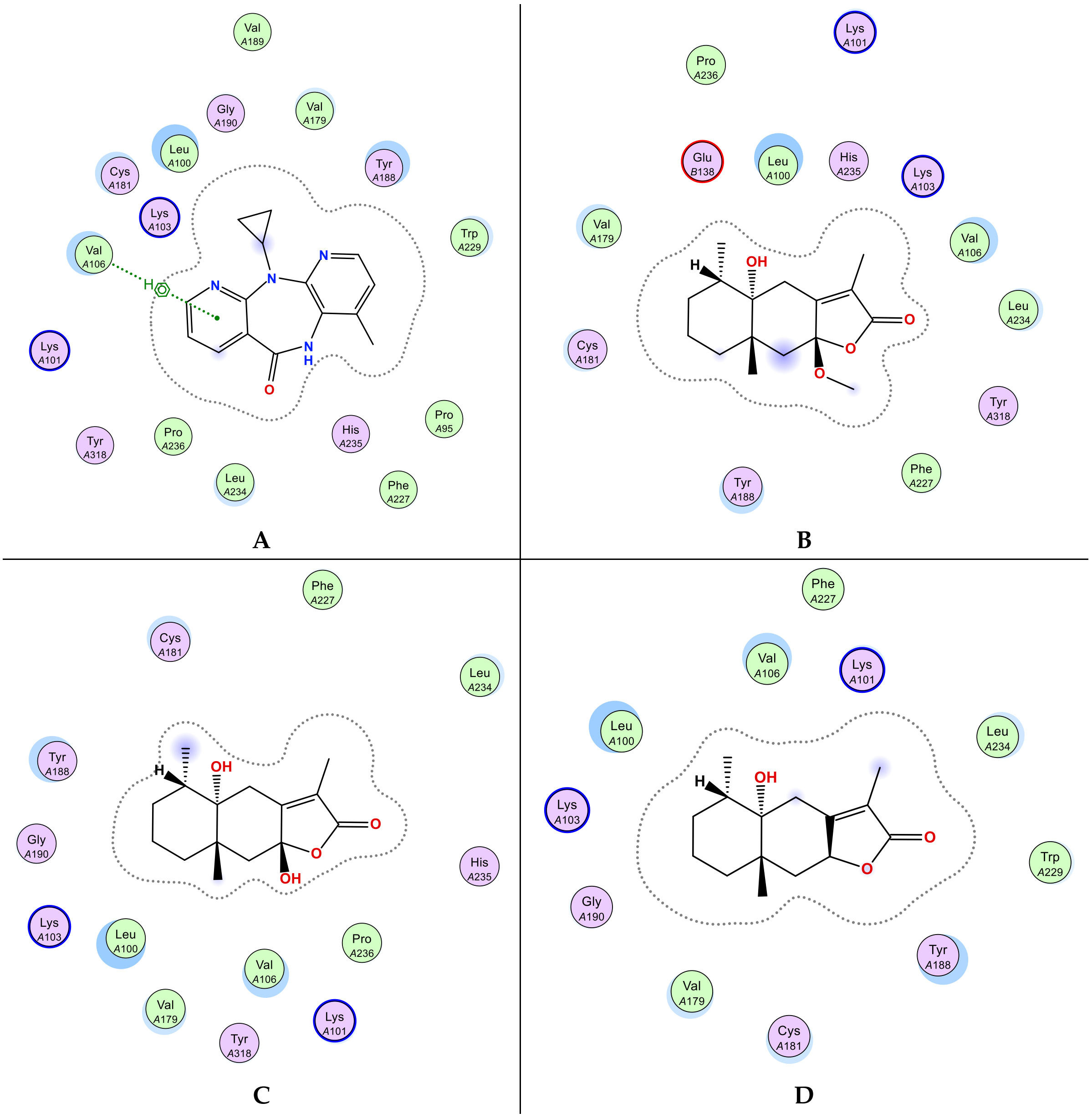

Figure S1: Results of Docking of compound 1-3 with HIV-Reverse Transcriptase (PDB ID: 1JLB); A-Nevirapine; B- compound **1**; C- compound **2**; D- compound **3**.

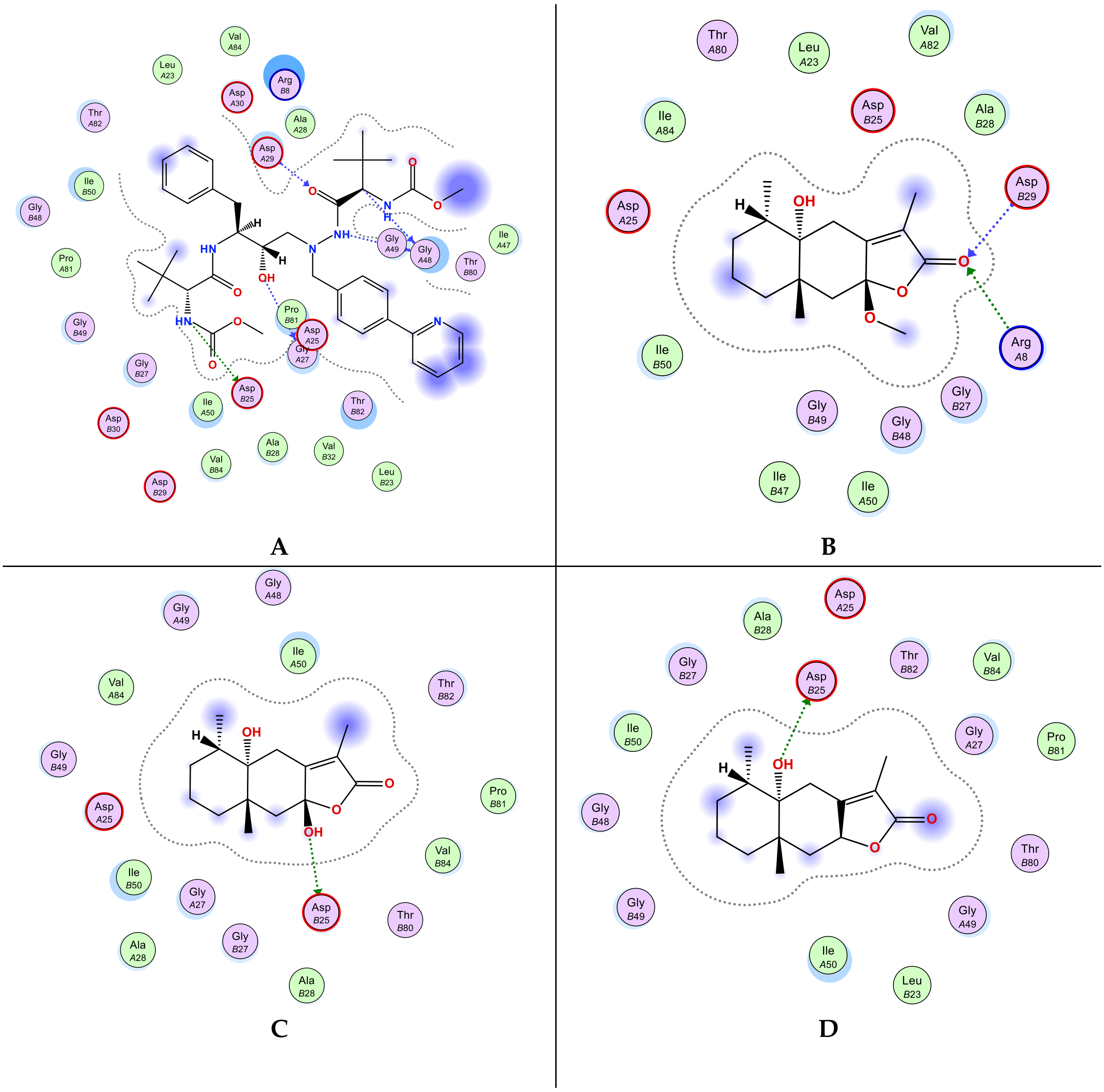

Figure S2: Results of Docking of compound 1-3 with HIV-1 Protease (PDB ID: 3EL9); A-Atazanavir; B- compound **1**; C- compound **2**; D- compound **3**.

Appendix 1 Mass Spectrum of 5β-hydroxy-8α-methoxy eudesm-7(11)-en-12, 8-olide (ermiasolide A) (**1**)

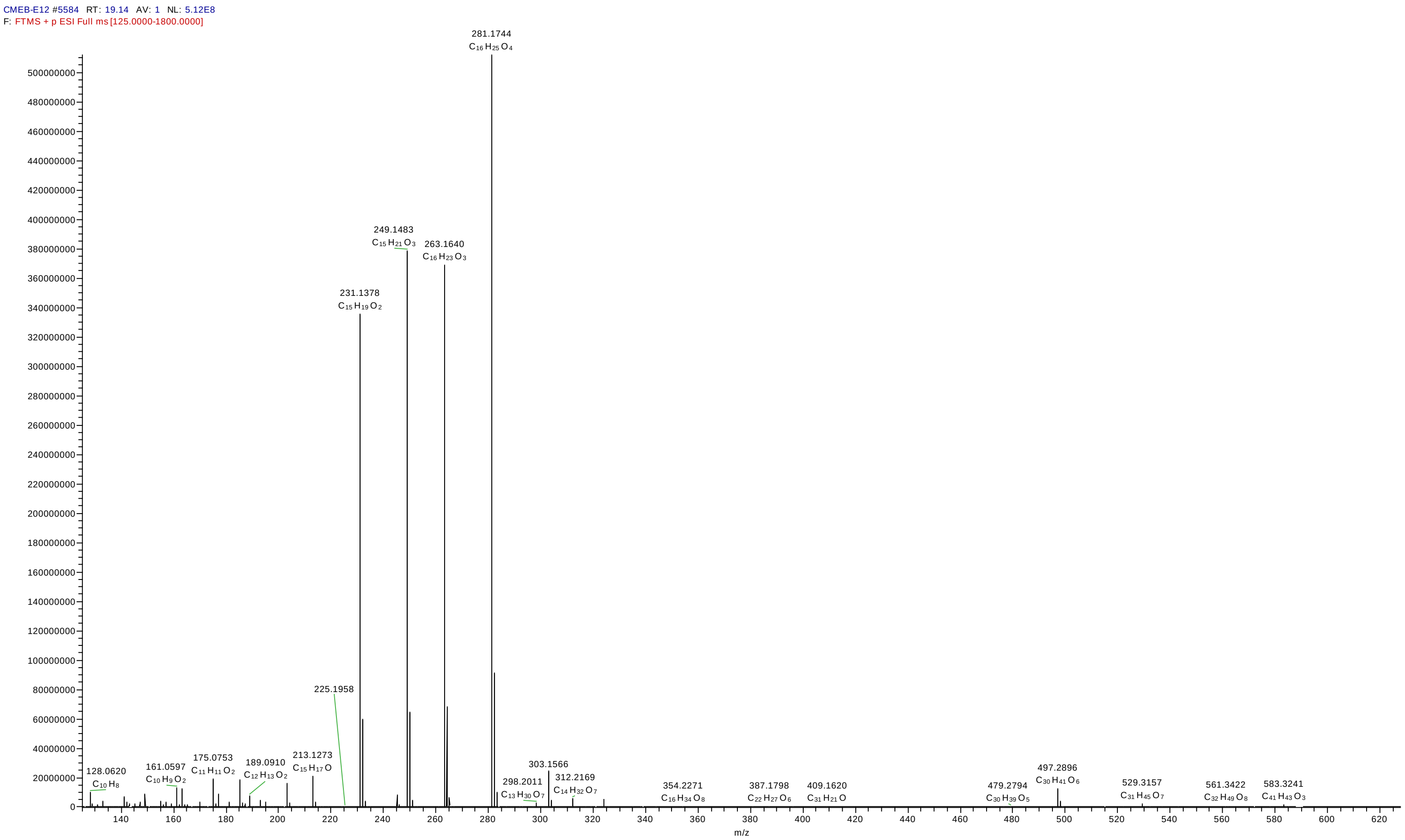

HRESIMS *m/z* 281.1744 [M+H]$^+$ (calcd. for $C_{16}H_{24}O_4$ + H, *m/z* 281.1753)

Appendix 2 ^{1}H NMR Spectra for 5β-hydroxy-8α-methoxy eudesm-7(11)-en-12,8-olide (ermiasolide A) (**1**)

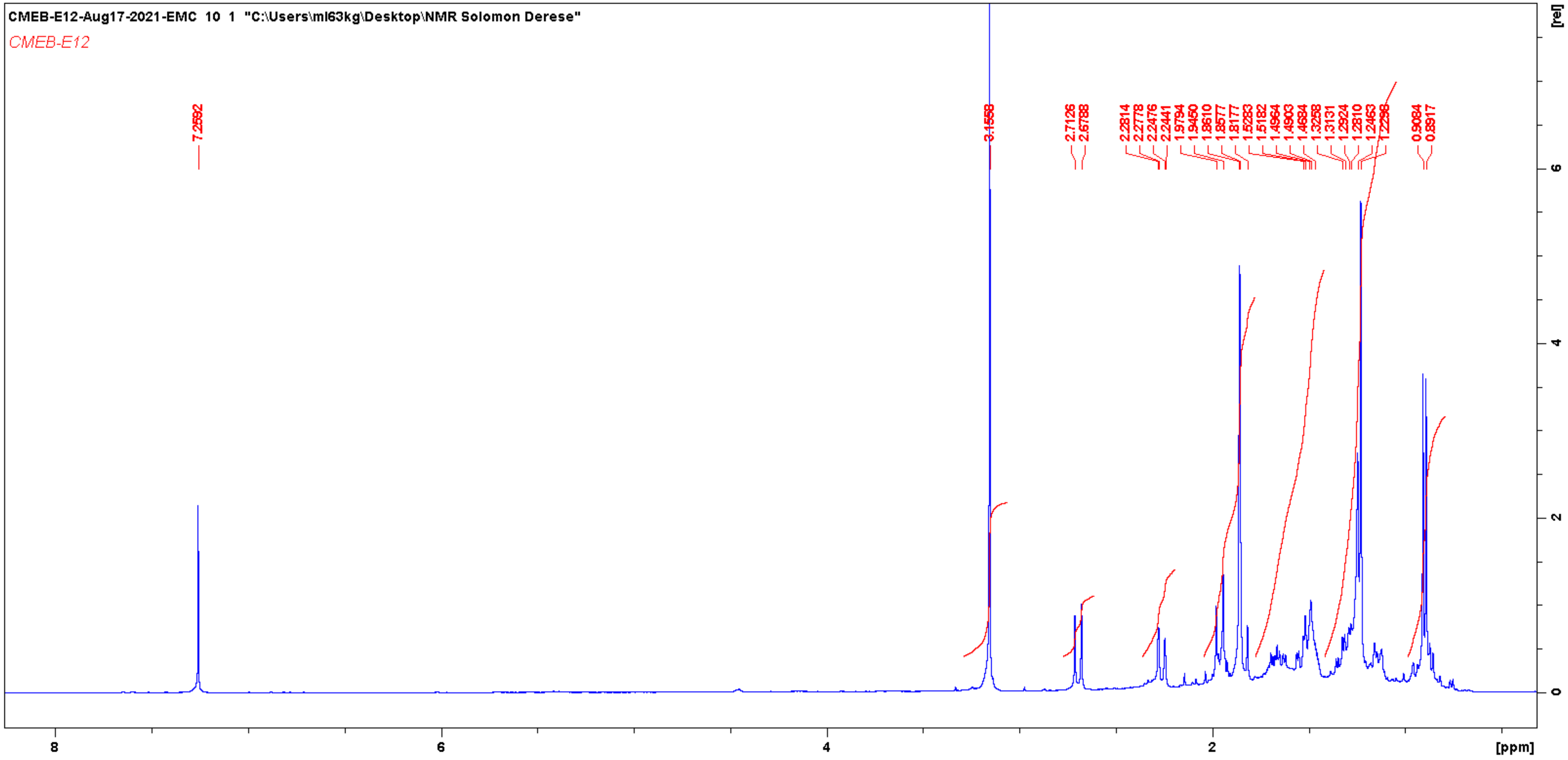

Appendix 3 ^{13}C NMR Spectra for 5β-hydroxy-8α-methoxy eudesm-7(11)-en-12, 8-olide (ermiasolide A) (**1**)

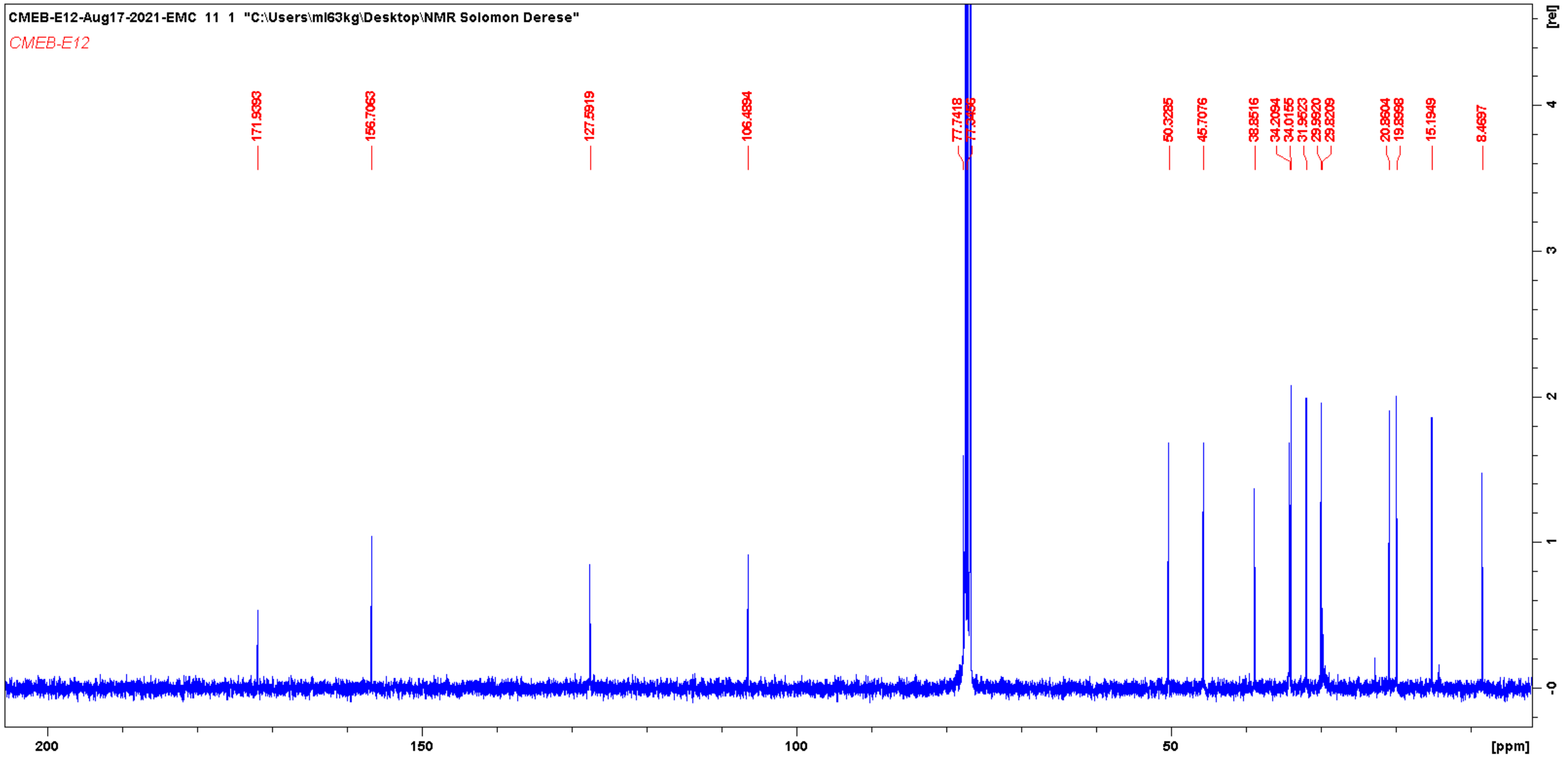

Appendix 4 DEPT Spectrum for 5β-hydroxy-8α-methoxy eudesm-7(11)-en-12, 8-olide (ermiasolide A) (**1**)

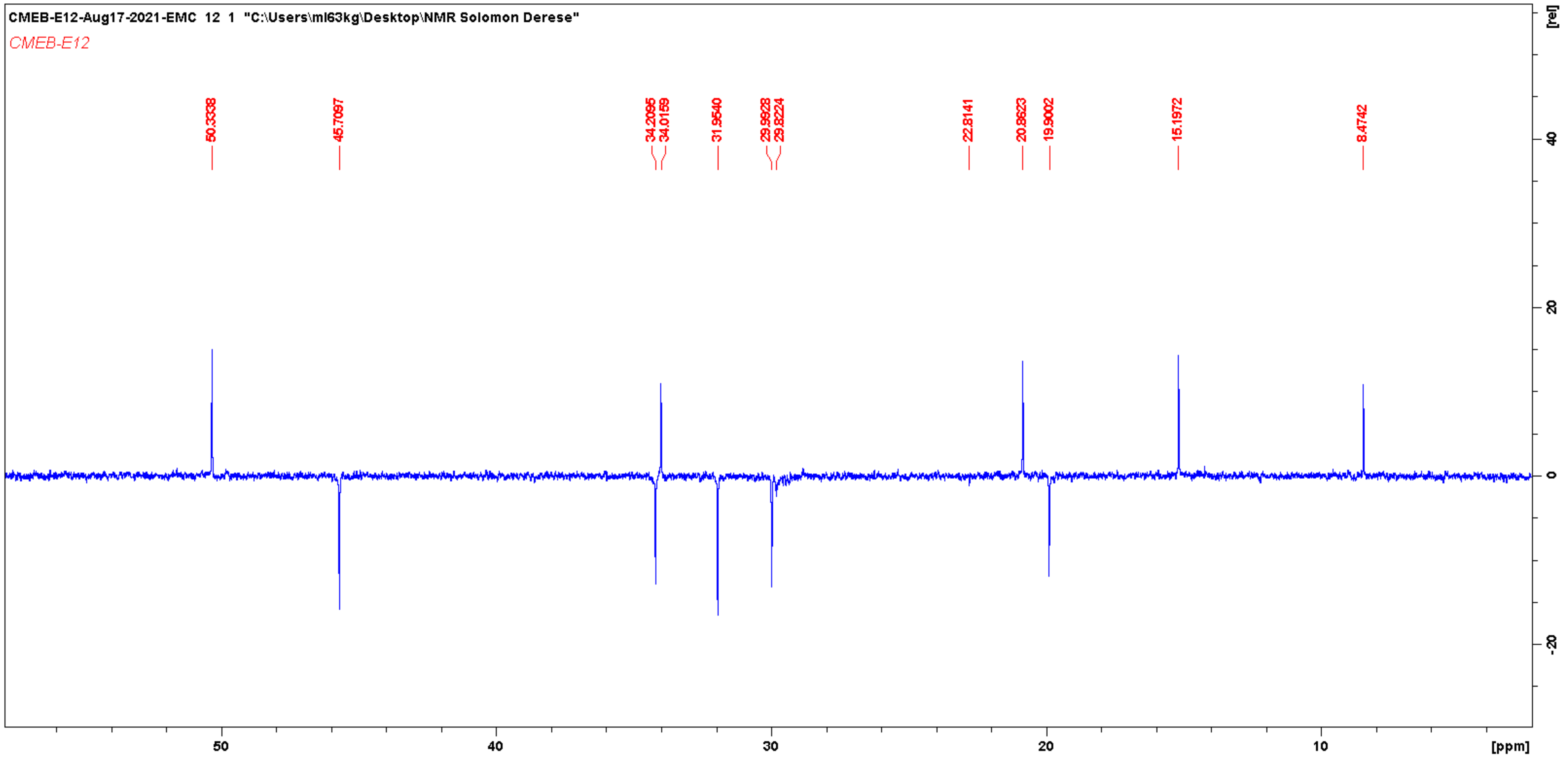

Appendix 5 HSQCDEPT Spectra for 5β-hydroxy-8α-methoxy eudesm-7(11)-en-12,8-olide (ermiasolide A) (**1**)

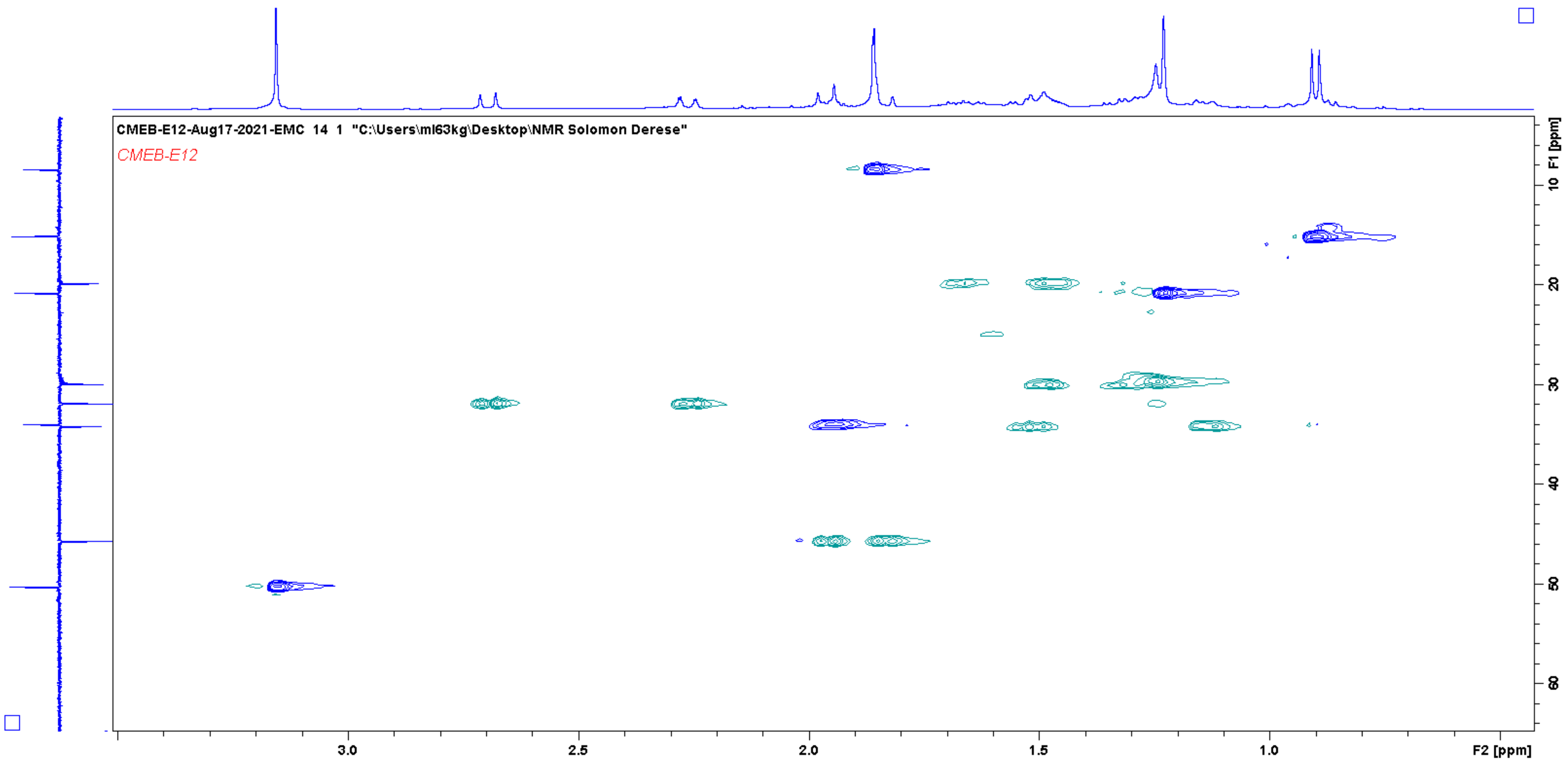

Appendix 6 HMBC Spectrum for 5β-hydroxy-8α-methoxy eudesm-7(11)-en-12, 8-olide (ermiasolide A) (**1**)

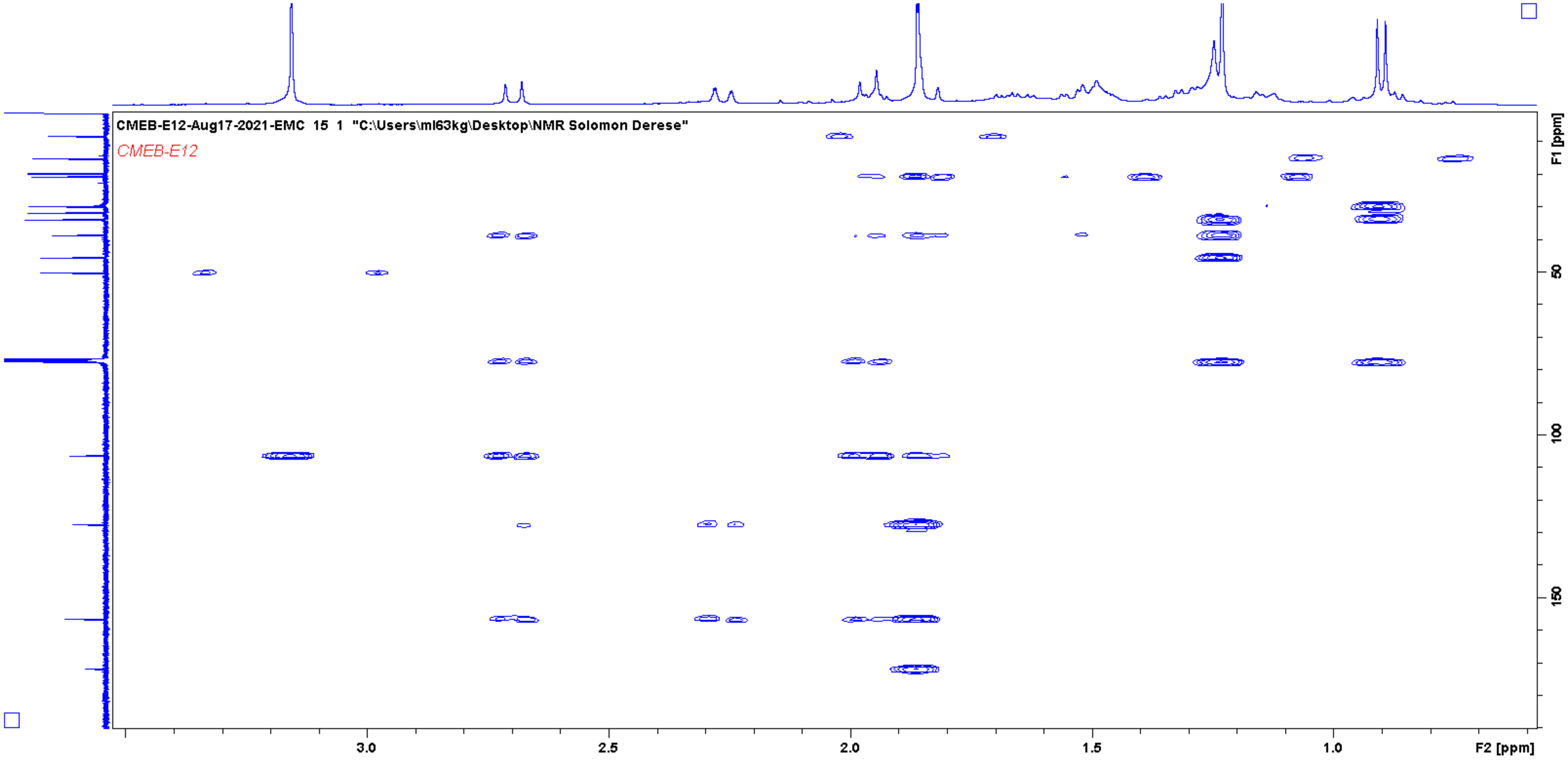

Appendix 7 COSY Spectrum for 5β-hydroxy-8α-methoxy eudesm-7(11)-en-12,8-olide (ermiasolide A) (**1**)

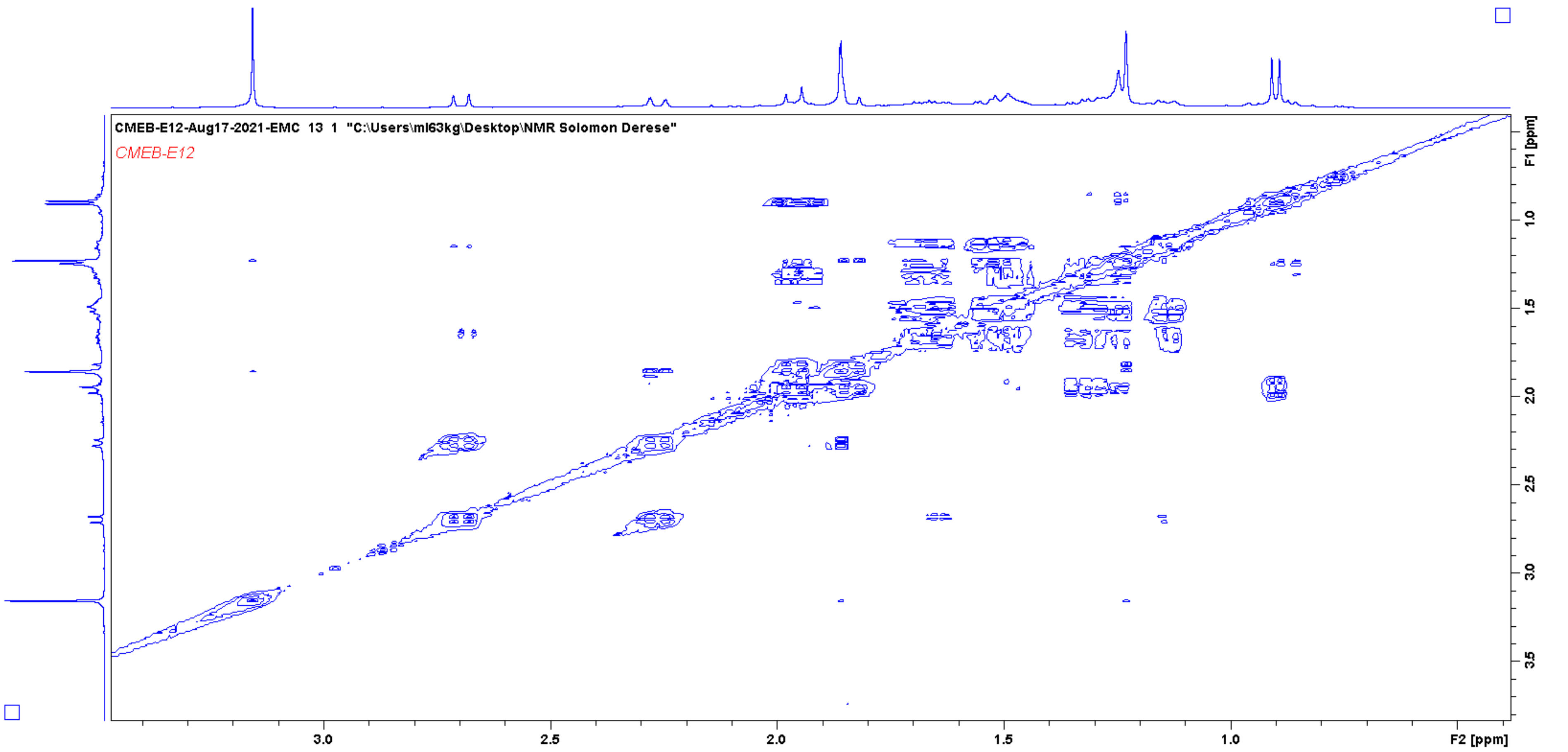

Appendix 8 NOESY Spectrum for 5β-hydroxy-8α-methoxy eudesm-7(11)-en-12,8-olide (ermiasolide A) (**1**)

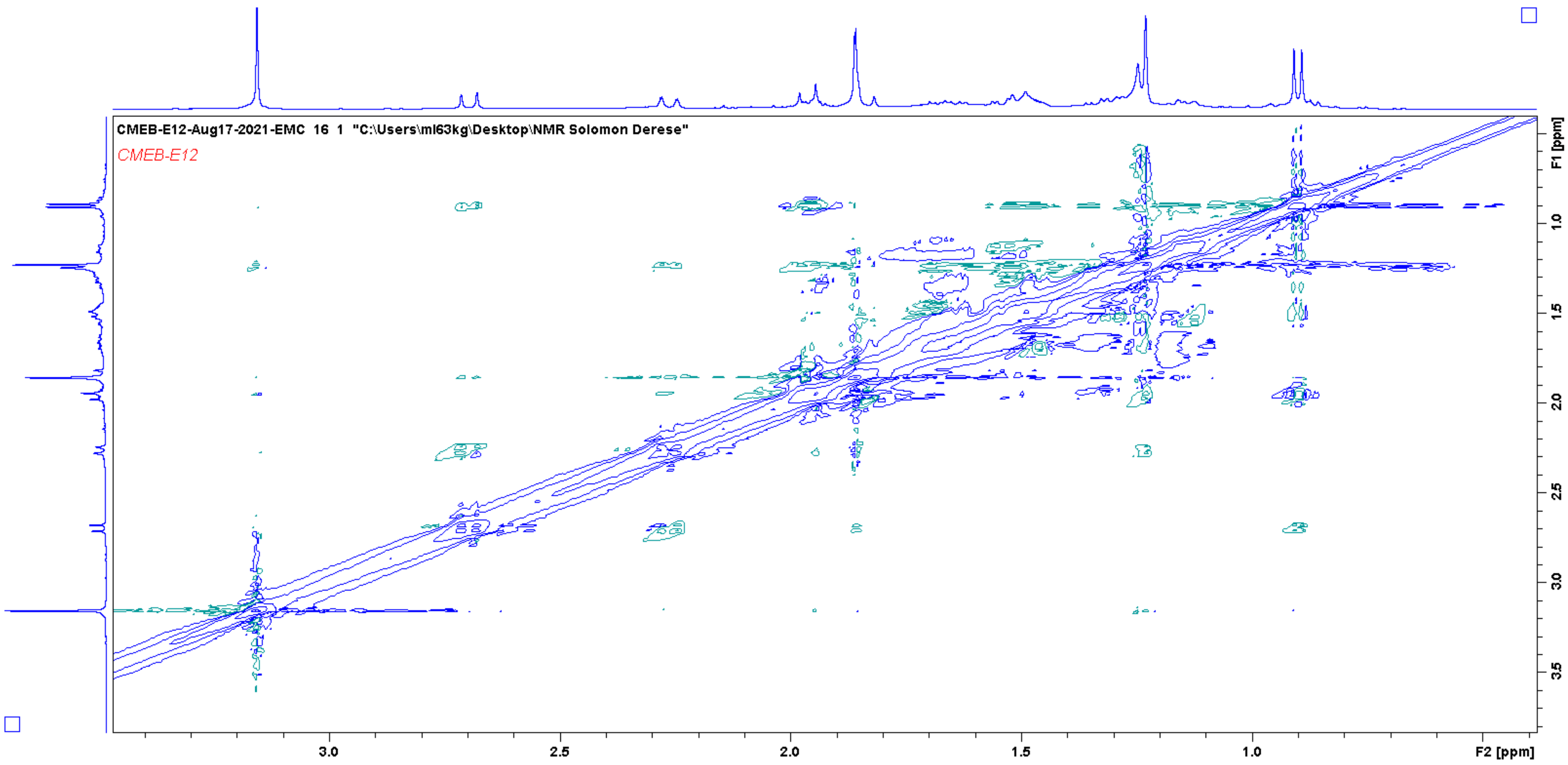

Appendix 9 Mass Spectrum of 5β,8α-dihydroxy eudesm-7(11)-en-12,8-olide (ermiasolide B) (**2**)

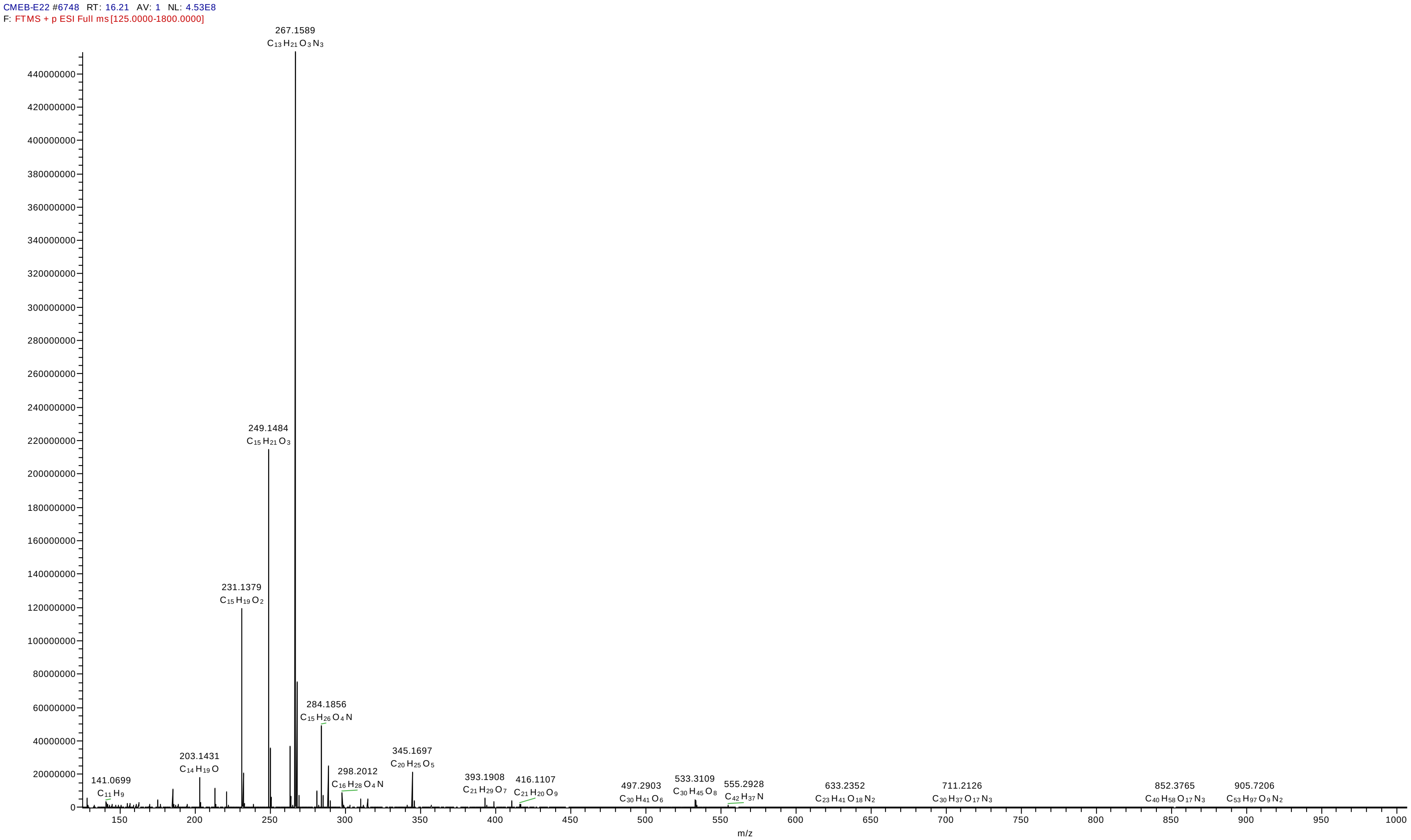

HRESIMS *m/z* 267.1589 [M+H]$^+$ (calcd. for $C_{15}H_{22}O_4$ + H, *m/z* 267.1591)

Appendix 10 ^{1}H NMR spectra of 5β,8α-dihydroxy eudesm-7(11)-en-12,8-olide (ermiasolide B) (**2**)

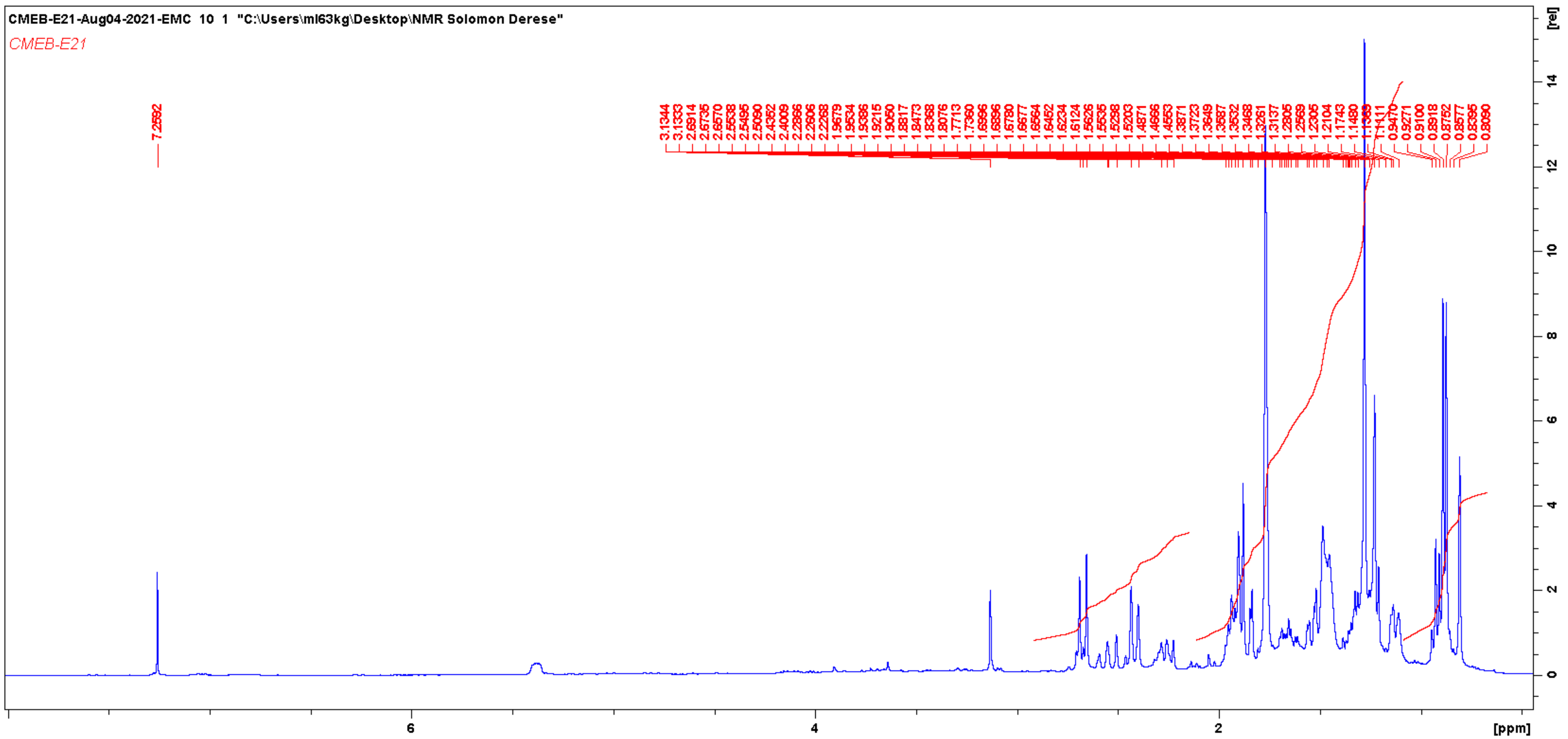

Appendix 11 ^{13}C NMR spectra of 5β,8α-dihydroxy eudesm-7(11)-en-12,8-olide (ermiasolide B) (**2**)

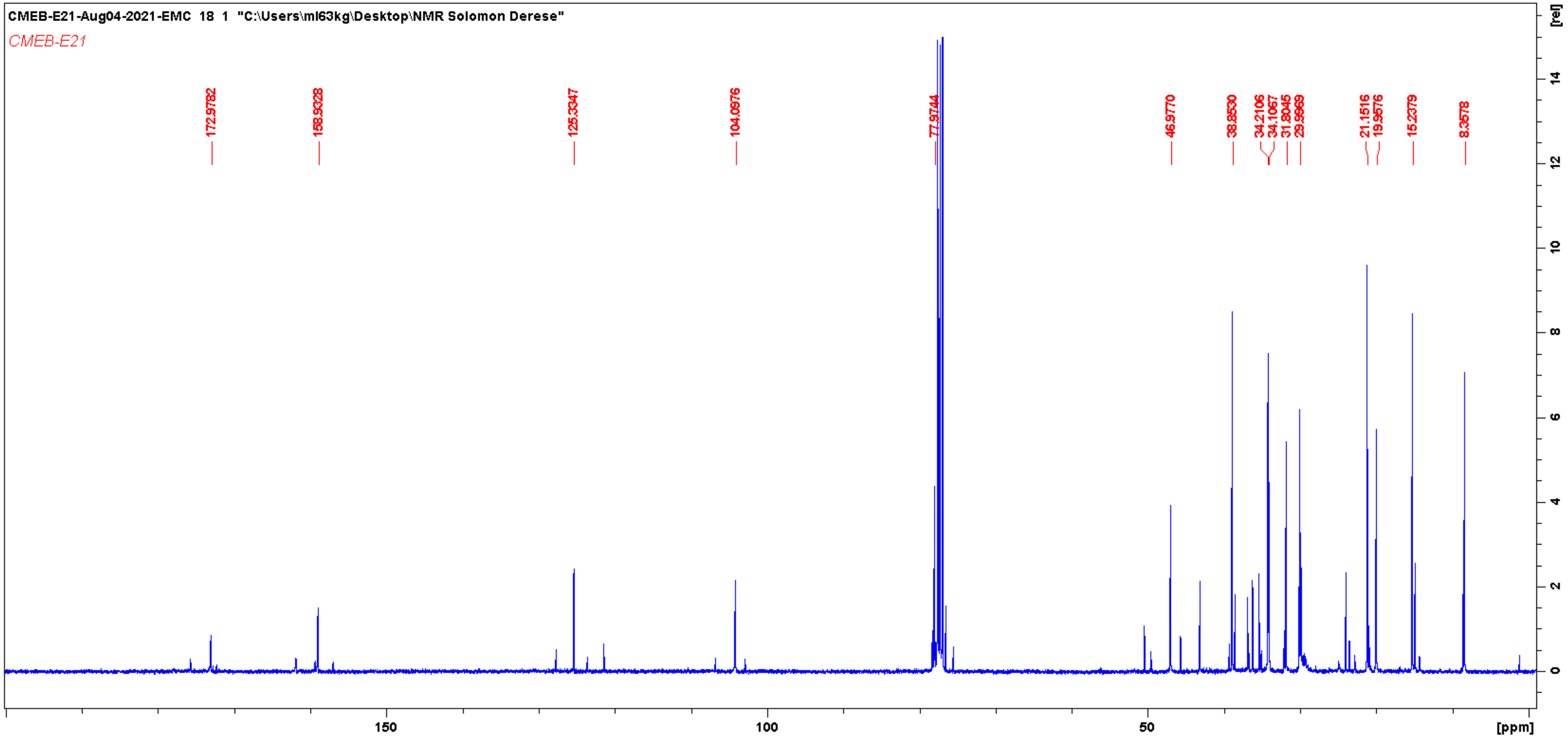

Appendix 12 DEPT spectrum of 5β,8α-dihydroxy eudesm-7(11)-en-12,8-olide (ermiasolide B) (**2**)

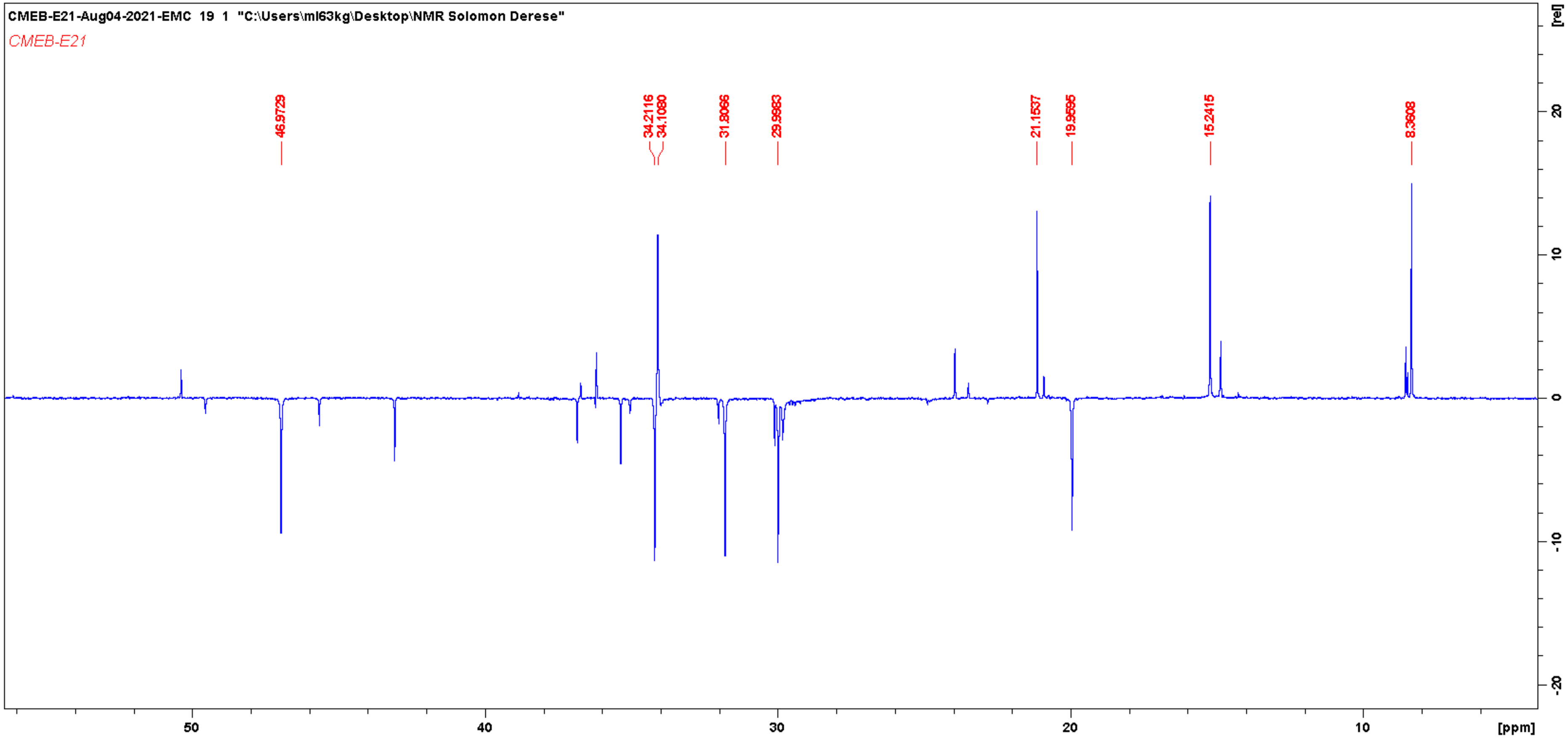

Appendix 13 HSQCDEPT spectrum of 5β, 8α-dihydroxy eudesm-7(11)-en-12,8-olide (ermiasolide B) (**2**)

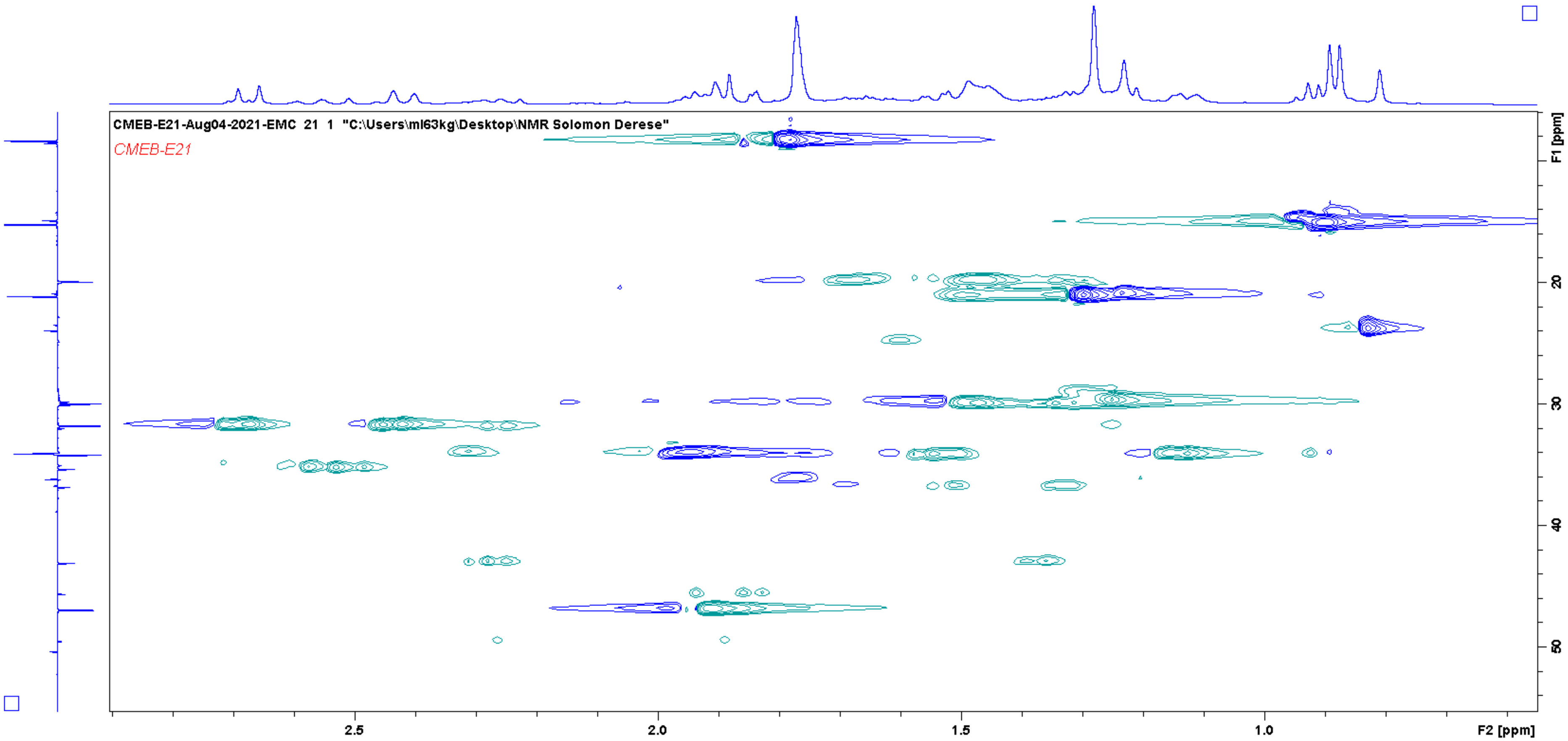

Appendix 14 HMBC spectrum of 5β,8α-dihydroxy eudesm-7(11)-en-12,8-olide (ermiasolide B) (**2**)

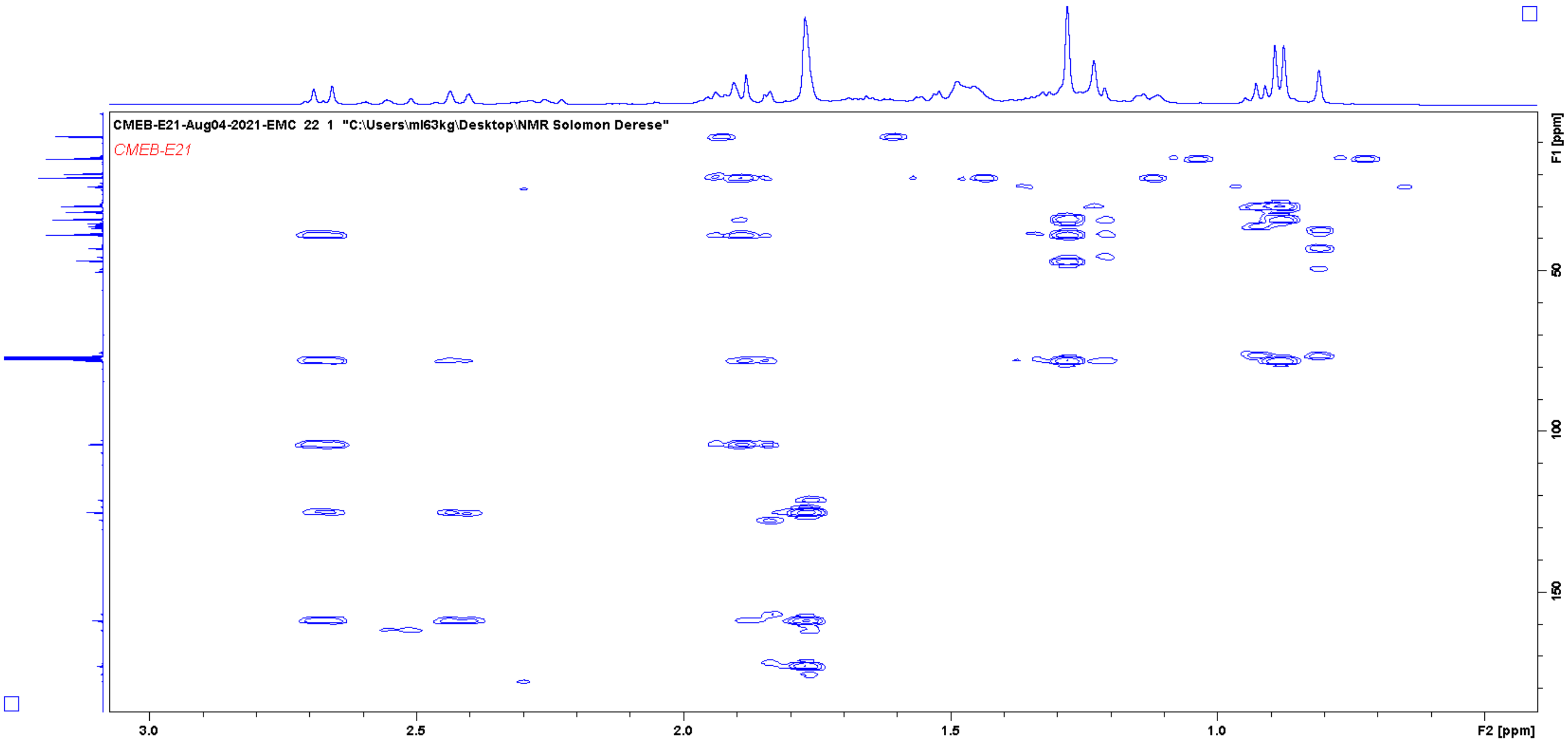

Appendix 15 COSY spectrum of 5β, 8α-dihydroxy eudesm-7(11)-en-12,8-olide (ermiasolide B) (**2**)

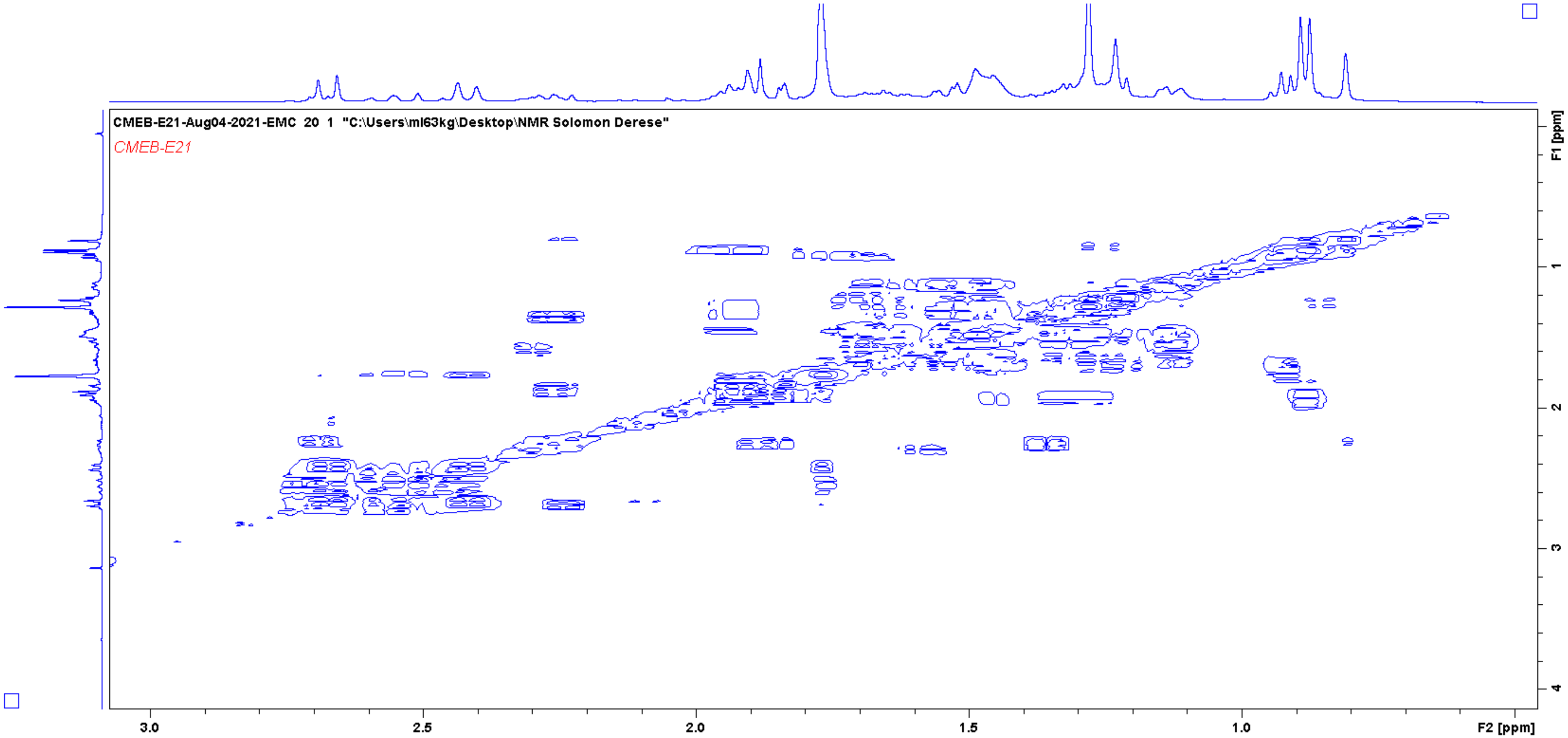

Appendix 16 NOESY spectrum of 5β, 8α-dihydroxy eudesm-7(11)-en-12,8-olide (ermiasolide B) **(2)**

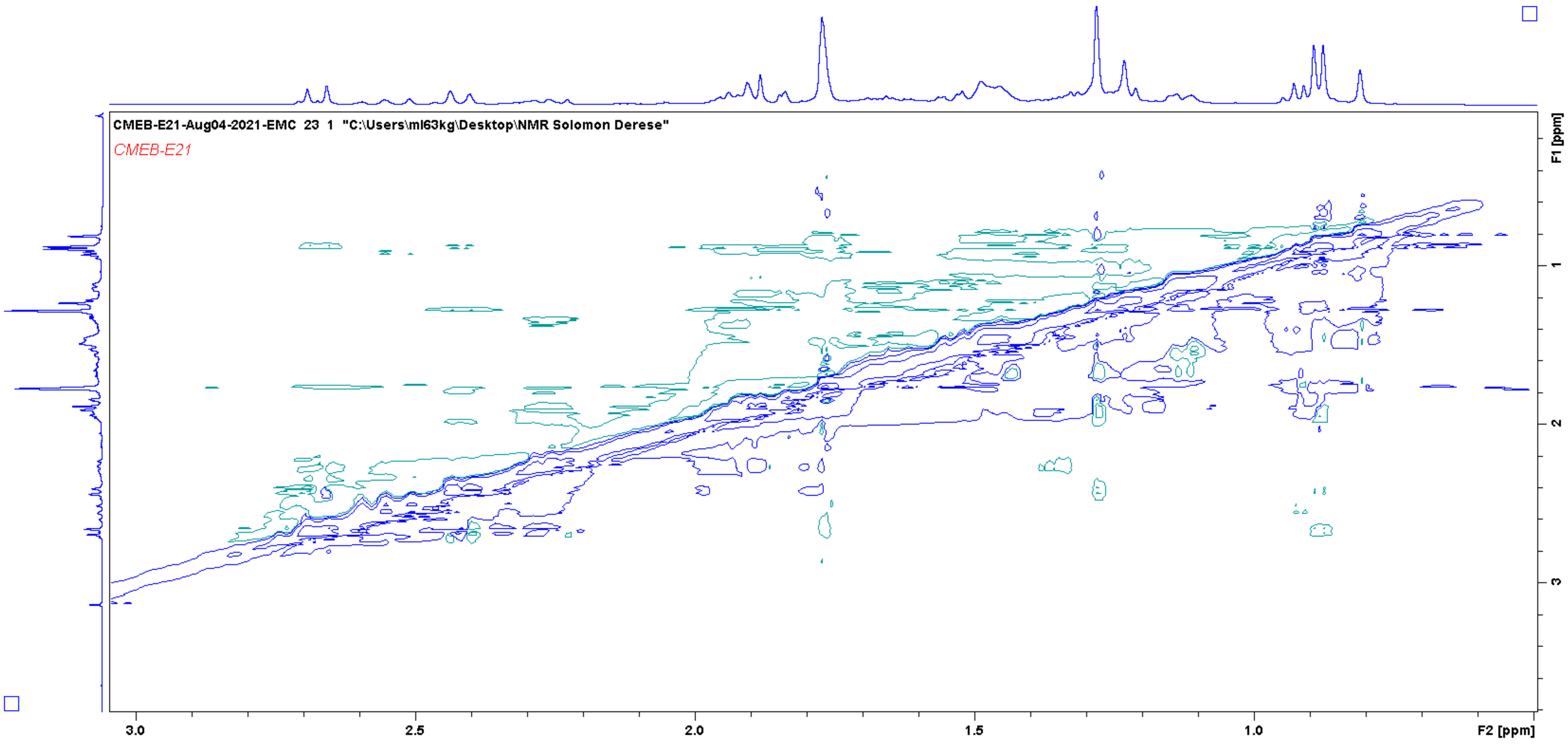

Appendix 17 Mass spectrum of 5β,8H-β-hydroxy eudesm-7(11)-en-12, 8-olide (ermiasolide C) (**3**)

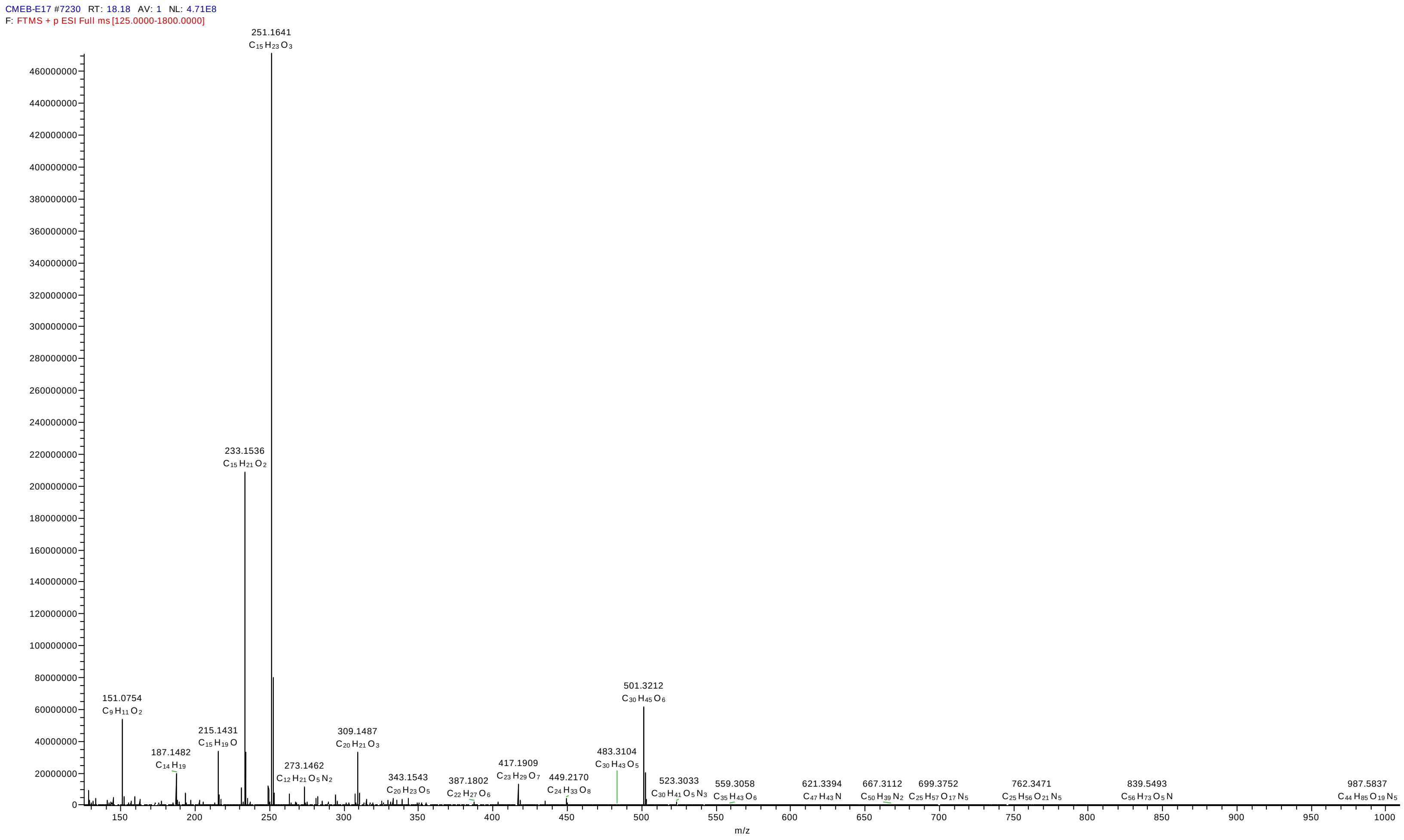

HRESIMS *m/z* 251.1641 $[M+H]^+$ (calcd. for $C_{15}H_{22}O_3$ + H, *m/z* 251.1642)

Appendix 18 ^{1}H NMR spectra of 5β,8H-β-hydroxy eudesm-7(11)-en-12, 8-olide (ermiasolide C) (**3**)

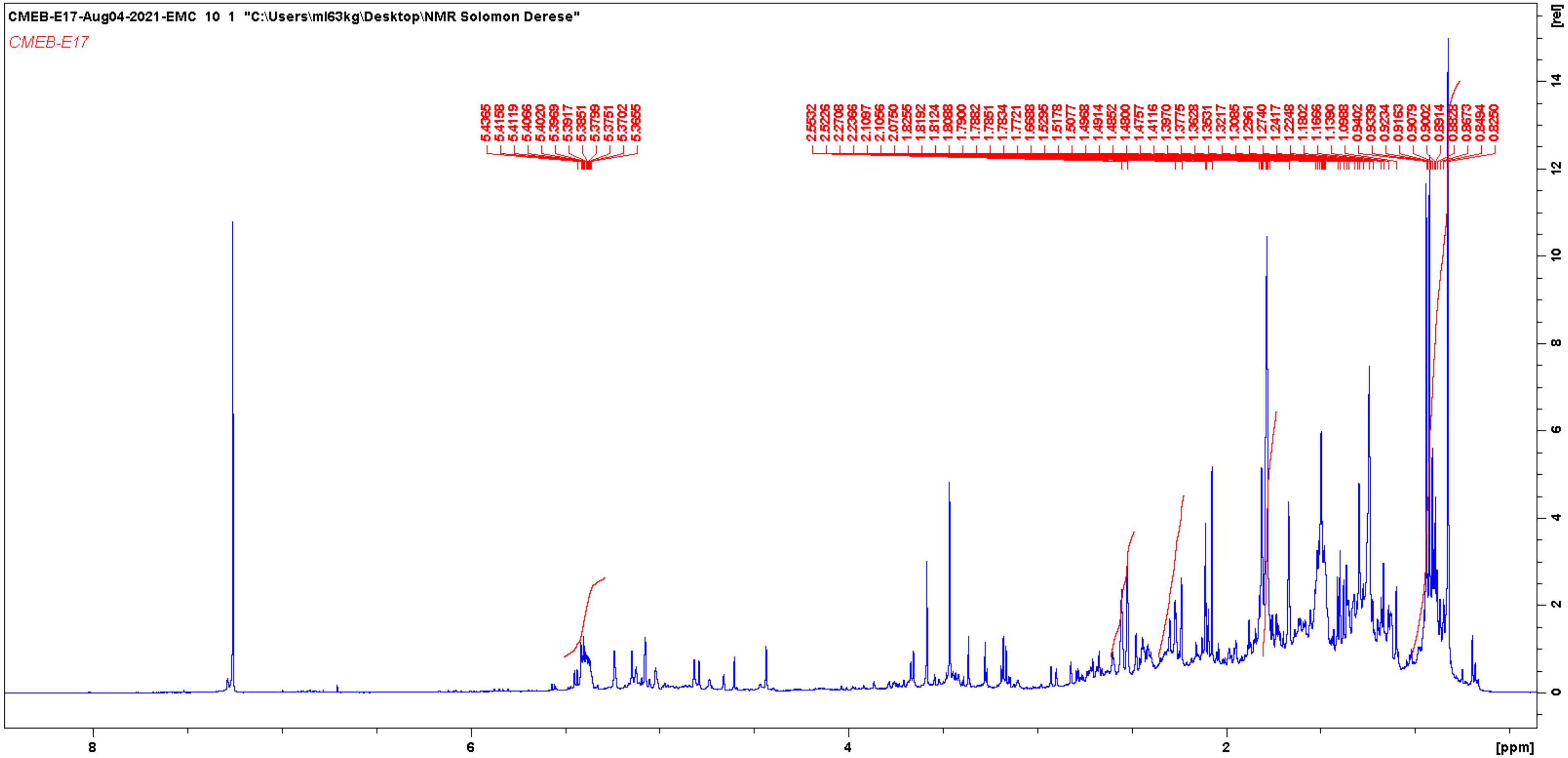

Appendix 19 ^{13}C NMR spectra of 5β,8H-β-hydroxy eudesm-7(11)-en-12, 8-olide (ermiasolide C) (**3**)

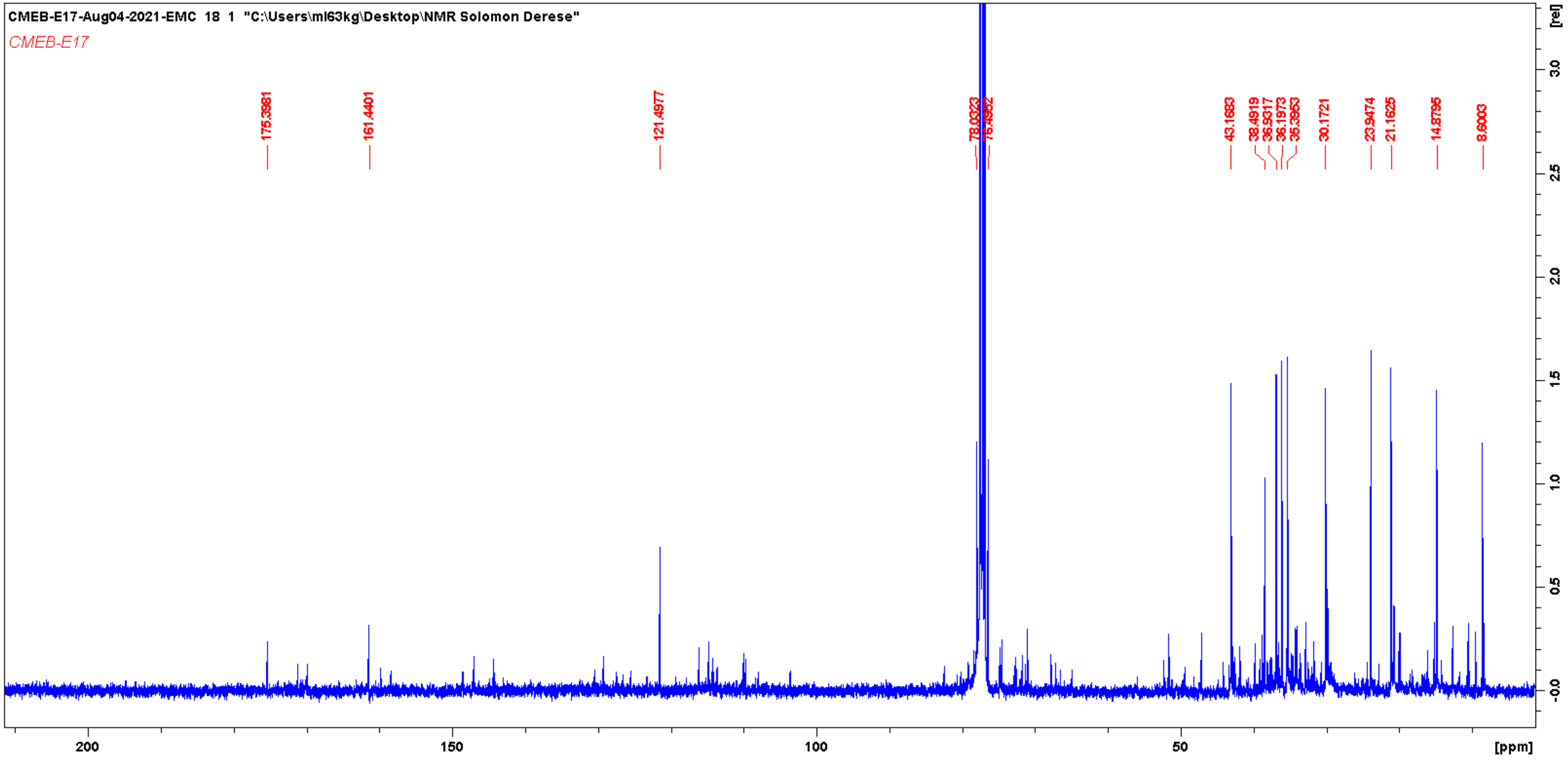

Appendix 20 DEPT spectrum of 5β,8H-β-hydroxy eudesm-7(11)-en-12, 8-olide (ermiasolide C) (**3**)

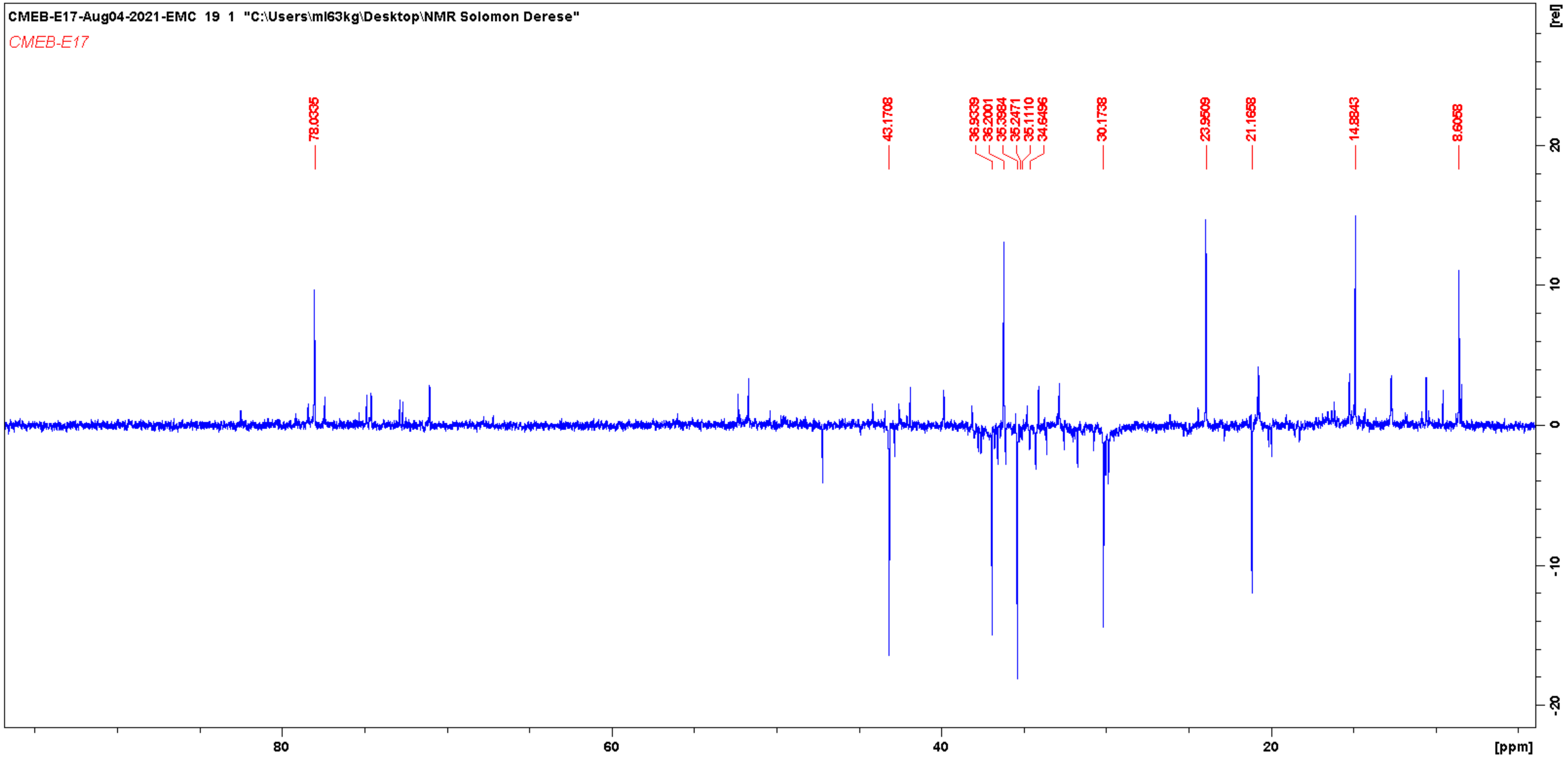

Appendix 21 HSQCDEPT spectrum of 5β,8H-β-hydroxy eudesm-7(11)-en-12, 8-olide (ermiasolide C) (**3**)

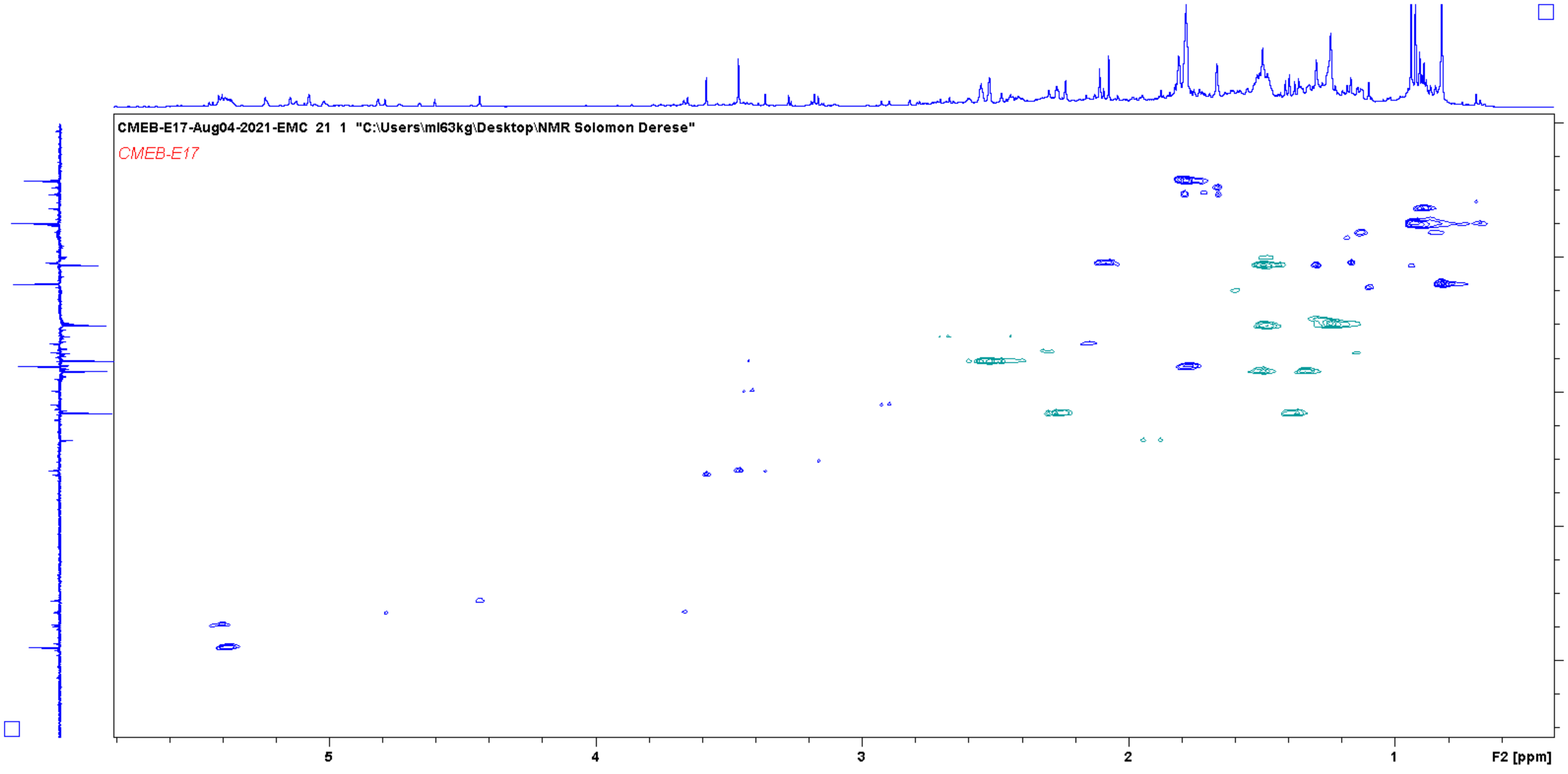

Appendix 22 HMBC spectrum of 5β,8H-β-hydroxy eudesm-7(11)-en-12, 8-olide (ermiasolide C) (**3**)

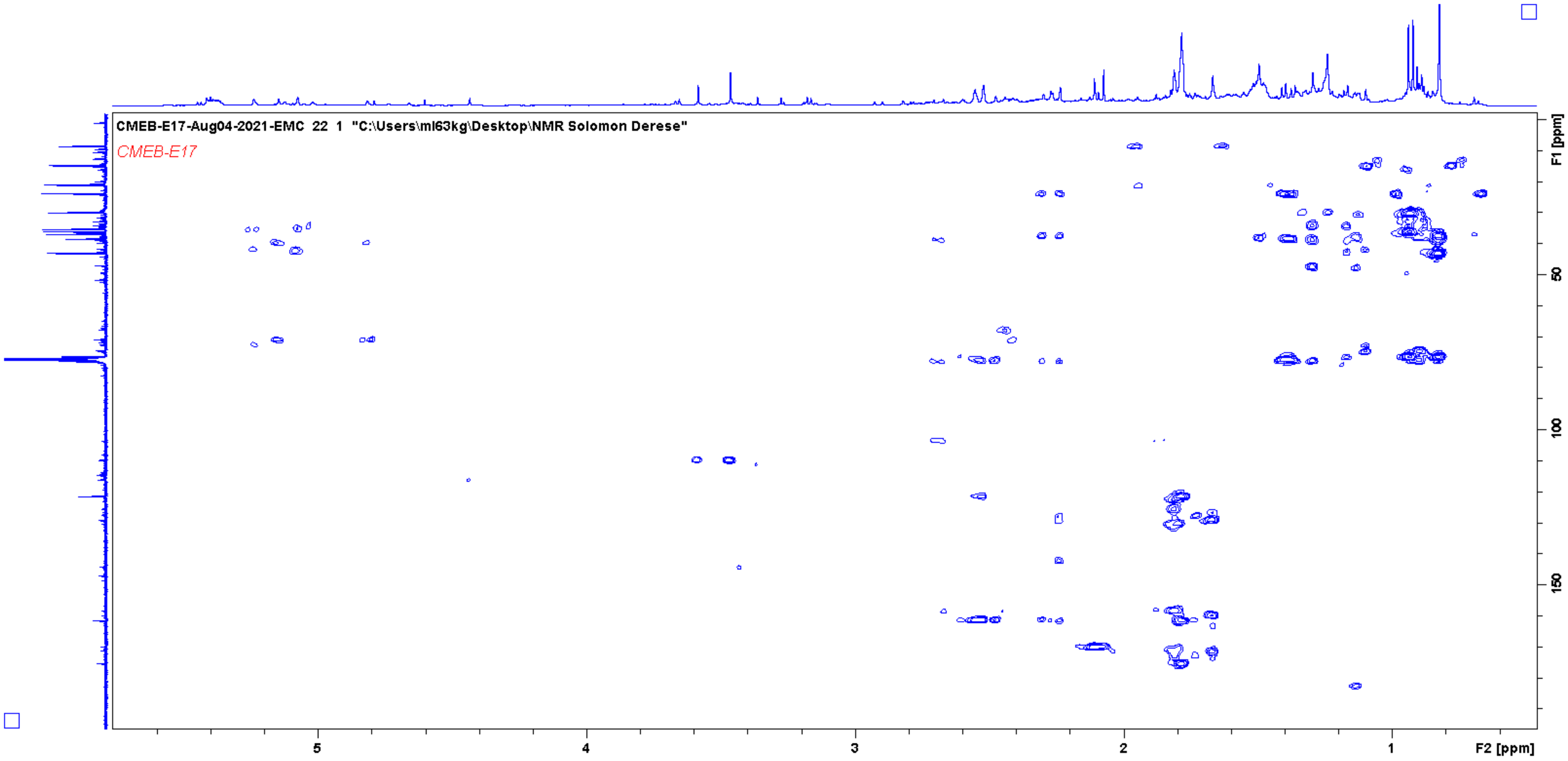

Appendix 23 COSY spectrum of 5β,8H-β-hydroxy eudesm-7(11)-en-12, 8-olide (ermiasolide C) (**3**)

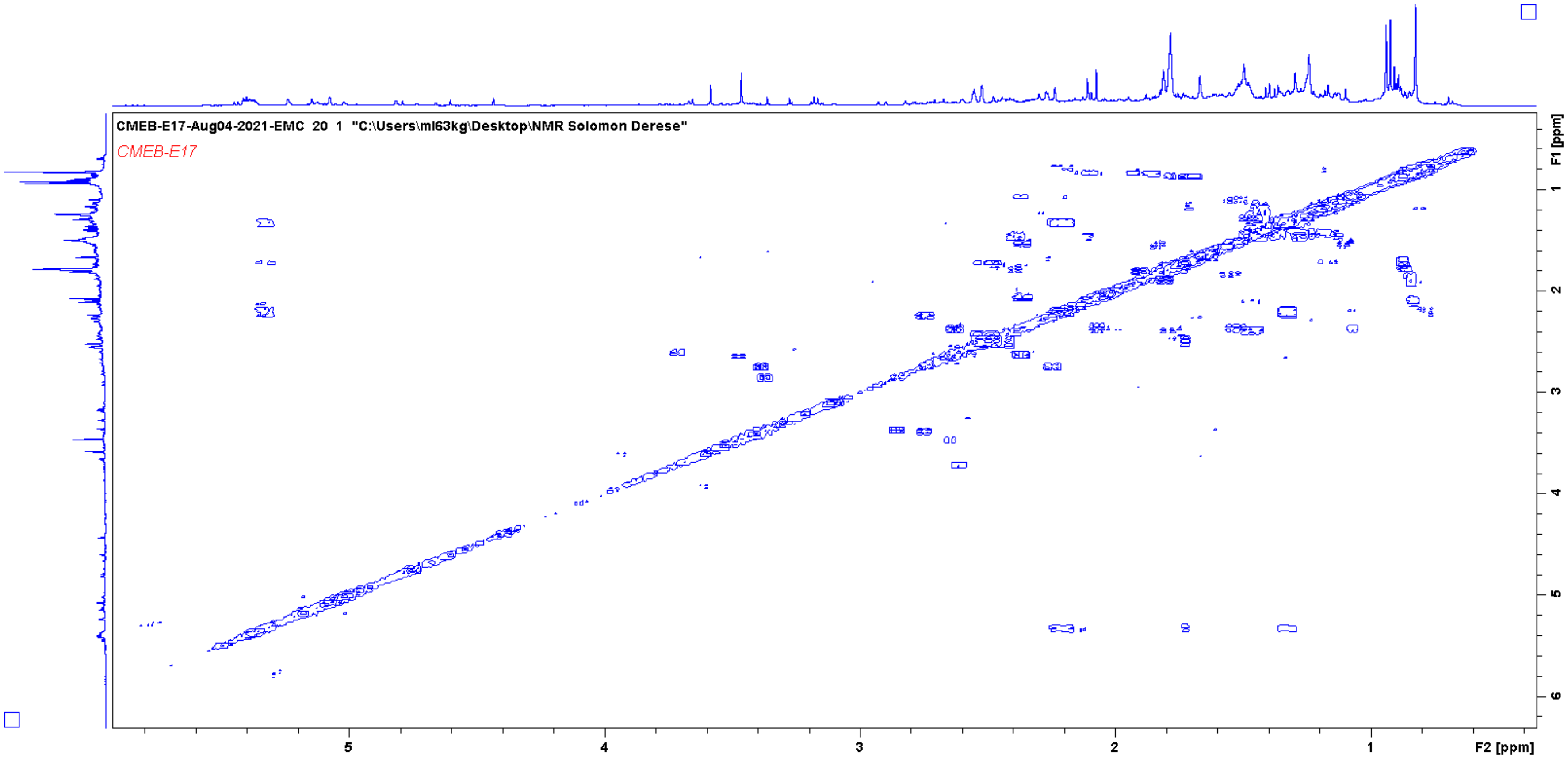

Appendix 24 NOESY spectrum of 5β,8H-β-hydroxy eudesm-7(11)-en-12, 8-olide (ermiasolide C) (**3**)

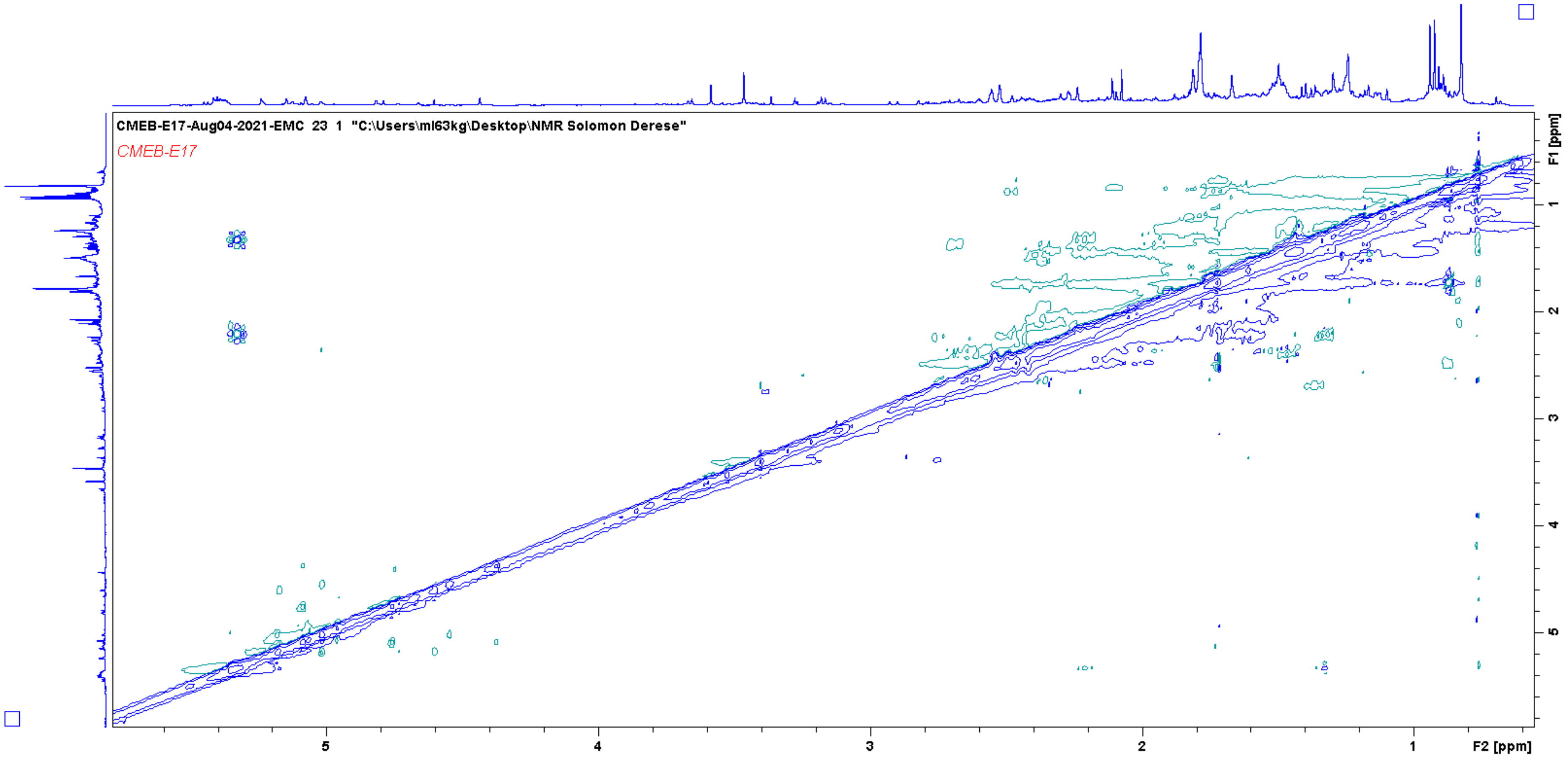

Supplement: Supplementary file 1 [file molecules-27-07040-s001.zip › molecules-1800300-Supplementary.pdf]
